# Identification of Photosynthesis-Associated C_4_ Candidate Genes through Comparative Leaf Gradient Transcriptome in Multiple Lineages of C_3_ and C_4_ Species

**DOI:** 10.1371/journal.pone.0140629

**Published:** 2015-10-14

**Authors:** Zehong Ding, Sarit Weissmann, Minghui Wang, Baijuan Du, Lei Huang, Lin Wang, Xiaoyu Tu, Silin Zhong, Christopher Myers, Thomas P. Brutnell, Qi Sun, Pinghua Li

**Affiliations:** 1 The Institute of Tropical Bioscience and Biotechnology, Chinese Academy of Tropical Agricultural Sciences, Hainan, Haikou, China; 2 Computational Biology Service Unit, Life Sciences Core Laboratories Center, Cornell University, Ithaca, New York, United States of America; 3 The Donald Danforth Plant Science Center, St. Louis, Missouri, United States of America; 4 State Key Laboratory of Crop Biology, College of Agronomy, Shandong Agricultural University, Tai’an, Shandong, China; 5 Partner State Key Laboratory of Agrobiotechnology, School of Life Sciences, The Chinese University of Hong Kong, Hong Kong, China; Institute of Crop Sciences, CHINA

## Abstract

Leaves of C_4_ crops usually have higher radiation, water and nitrogen use efficiencies compared to the C_3_ species. Engineering C_4_ traits into C_3_ crops has been proposed as one of the most promising ways to repeal the biomass yield ceiling. To better understand the function of C_4_ photosynthesis, and to identify candidate genes that are associated with the C_4_ pathways, a comparative transcription network analysis was conducted on leaf developmental gradients of three C_4_ species including maize, green foxtail and sorghum and one C_3_ species, rice. By combining the methods of gene co-expression and differentially co-expression networks, we identified a total of 128 C_4_ specific genes. Besides the classic C_4_ shuttle genes, a new set of genes associated with light reaction, starch and sucrose metabolism, metabolites transportation, as well as transcription regulation, were identified as involved in C_4_ photosynthesis. These findings will provide important insights into the differential gene regulation between C_3_ and C_4_ species, and a good genetic resource for establishing C_4_ pathways in C_3_ crops.

## Introduction

With growing population and increasing urbanization, humanity faces a looming food crisis, which to prevent, will require yields to be increased by at least 50% over the next 40 years [1, 2]. In addition, extreme climate changes, decreasing availability of water and energy resources, and competitions between grains for bio-fuels and food could worsen the situation. One of the most promising solutions is to introduce the C_4_ photosynthetic pathways into C_3_ crops such as rice and soybeans, to improve their water, radiation, and nitrogen use efficiency [1–3], resulting in higher yields than present day C_3_ crops [4].

In the past few decades, numerous efforts have been made to introduce C_4_ traits into C_3_ plants, for example the work carried out by the C_4_ Rice Consortium (http://irri.org/c4rice). Although none of them have so far demonstrated significantly enhanced photosynthetic properties in transgenic plants, the expression patterns and activities of many known C_4_ associated genes and proteins were thoroughly studied, and can be used as the basis for future engineering attempts [5–7]. Several features of studied C_4_ genes are: (1) The C_4_ pathway independently evolved more than 60 times from C_3_ plants [8]. Orthologues of genes encoding classic C_4_ enzymes preexisted in their C_3_ ancestors but are usually lowly expressed in C_3_ plants, while in C_4_ plants these genes are highly expressed and co-regulated by multiple stimuli, e.g., light [5, 9]; (2) Many proteins, encoded by multi-gene families and thought to fulfill housekeeping functions in C_3_ species [7, 9], are recruited into the C_4_ pathway after a neo-function is acquired for the C_4_ paralog [10], which may change its gene expression pattern [11]; (3) C_4_ genes are often expressed in a cell-type specific manner, i.e. bundle sheath (BS) or mesophyll (ME) cells. These characteristics could be exploited to identify novel C_4_ genes as well as their regulatory networks, which in practice could provide guidance for strategies of establishing the C_4_ cycle in C_3_ plants, e.g., by transferring a group of genes instead of a single gene into C_3_ crops [5].

With recent advances in sequencing technologies, genome assemblies of multiple C_4_ species including maize [12], sorghum [13] and new C_4_ model species, foxtail millet (*Setaria Italica*) [14, 15], are currently available, providing a good opportunity to dissect the C_4_ pathway using system biology approaches. To date, several transcriptomics and proteomics studies have provided insight into C_4_ gene expression and protein accumulation by comparative analysis of BS and ME cells in maize [16–21], green foxtail (*Setaria viridis*) [22], and rice [23], transcriptional profiling along a leaf development gradient in maize [24, 25] and between maize and rice [26], and between both distantly and closely related C_3_ and C_4_ species [27, 28]. Hundreds of differentially accumulated genes and proteins were identified and functionally characterized. Little, however, is known about the downstream regulatory networks of genes and protein interactions responsible for the fundamental anatomical features in both C_4_ and C_3_ species [29], as well as the mechanisms controlling the expression and function of well characterized C_4_ genes[9]. Systems biology analysis of multiple lineages of C_3_ and C_4_ species [7, 9], and comparative studies across species could provide great promise for identifying unknown genes that control many, yet unknown, C_4_ functions [30]. Recently, novel cell type-specific cis-regulatory elements and candidate transcription factors of C_4_ photosynthesis have been identified, by comparing sets of leaf gradient transcriptome data from maize and rice [26]. In that study, transcriptome data from anatomically and developmentally different leaf sections of maize and rice were projected to a unified gradient to facilitate cross-species clustering analysis on orthologue gene sets. The limitation of this approach is that when studying tissues from two species that are very divergent, it is not always feasible to project a unified gradient.

Gene co-expression network analysis, which uses transcriptomic data (either microarray or RNA-seq data) to group genes according to the similarity of their expression profiles [31], is one of the most powerful methods to explore genes relationships and to predict their function. It is presumed that genes that exhibit similar expression profiles across various tissues/samples are often functionally related [32]. The identified groups are referred to as modules, while the gene relationships within groups are referred to as networks, where nodes represent genes, and edges represent the correlations between pairs of genes [31, 33]. In plants, this method has been successfully applied many times to identify new members of biological processes [34–36]. When multiple species data are available, this process can be refined by extracting the gene co-expression networks found independently in each species, as biologically irrelevant associations caused by noise are not likely to be repeatedly observed in the co-expression networks in different species [32]. Two different strategies can be used for multi-species comparisons: (1) To find conserved modules across species with common gene orthologues [37] and then compare their expression patterns and expression levels, (2) to detect differentially co-expressed modules in which gene orthologues show different network structures between species.

In the present study, expression profiles of segments along a leaf developmental gradient [29], was used to conduct gene co-expression analysis in one C_3_ and three C_4_ species. The goal of our study was to identify C_4_ candidate genes, which are light-regulated and functionally different between C_4_ and C_3_ species (e.g., different expression levels, different expression patterns, present in C_4_ species but absent in C_3_ species), through genome-wide transcriptome data comparisons. Such C_4_ candidate genes may be used in the future as a useful source for engineering C_4_ traits into C_3_ crops for production improvement.

## Material and Methods

### Plant material

In total, leaf gradient transcription data from four species, including one C_3_ (rice) and three C_4_ (maize, green foxtail and sorghum), were used in this study. Of which, 15 sections of maize (*Zea mays*, inbred B73) and 11 sections of rice *(Oryza sativa* var. Nipponbare) data were derived from Wang et al. [26], and 13 sections of sorghum (S*orghum bicolor* var. BTx623) and 10 sections of green foxtail (*Setaria viridis*, ecotype A10.1) data, which derived in each case from 10-day-old third leaves were generated. The growth conditions of these four species were described previously [24, 26], in detail, under an 80:20 mix of metal halide, with capsylite halogen lamps at light intensity of 550 μmol/m^2^/sec, 12:12 L/D, 31°C L/22°C D and 50% relative humidity. All samples were harvested three hours after light on in the morning, pooled from at least seven plants (seven for maize, rice and sorghum, twenty for green foxtail) per biological replicate and have at least three biological replicates (five for maize, four for rice and three for sorghum) except green foxtail, which has only one replicate. We believed the data from green foxtail is reliable based on following reasons: (1) the samples of green foxtail showed very similar clustering patterns as maize and sorghum throughout all the sections (S1 Fig); (2) the expression patterns of classical C_4_ genes (e.g., PPDK, NADP-MDH, NADP-ME), which were used as case control in our study, were the same as maize and sorghum; (3) additional two C_4_ species (maize and sorghum) were also used, the cross species validation designed in the study can minimize the false-positive results due to lack of replication in green foxtail.

### Sequence analysis

Total RNA was extracted using TRIzol (Invitrogen, CA) following the manufacturer's suggestion from four species and subsequent RNA-seq libraries were constructed according to Wang et al. [24, 26]. 169M, 332M, 141M and 364M raw reads were generated by single end 35 bp, 51 bp, 51 bp and 35 bp sequencing with Illumina HiSeq 2000 machine from maize, sorghum, green foxtail and rice. After sequence quality examination, reads were mapped to the reference genomes (B73_AGPv2 for maize via MaizeSequence.org, rice_v6 for rice via rice.plantbiology.msu.edu, and JGIv2.0.21 for green foxtail and Sorbi1.22 for sorghum via plants.ensembl.org) using Tophat v2.0.10 [38] with most default settings (e.g., mismatches = 2, threads = 6) but without novel junctions detection (—no-novel-juncs). Because green foxtail reference genome is not currently available, the reads were mapped to its domesticated cultivar, foxtail millet (*setaria italica*). Reads counting and calculation of RPKM were described previously [26], and gene expression level was finally expressed as mean of RPKM across replicates. The reliability of RNA-seq was validated by qPCR ([39], S1 Table).

Before gene co-expression network analysis, low expressed loci were filtered by using RPKM > 1 in more than 10% sections (i.e., genes with RPKM > 1 in more than 2 out of 15 maize, 13 sorghum, 10 green foxtail and 11 rice sections are kept), and outliers were detected by clustering of samples with the correlation of gene expression [39]. Section 1, harvested from leaf base that displays very different expression profiling from other sections in three C_4_ species, was detected as outlier and removed from the analysis to avoid the network structures being dominated by difference between section 1 and the others.

### Gene co-expression network analysis

Gene co-expression networks were constructed by WGCNA [33] in each species respectively. In order to render the network scale free, different soft thresholding powers (e.g., 10 for maize, 12 for green foxtail, 18 for sorghum and 16 for rice) were chosen to transform Pearson similarity matrix into an adjacency matrix. Modules were determined by the dynamic tree cut method, and modules with high correlated genes (e.g., Pearson correlation > = 0.9) were merged. Modules were named as the first upper letter of each species then followed by their module colors, e.g., M.red and R.black represent red module in maize and black module in rice, respectively. Functional categories enrichment was conducted as previously described [24] based on MapMan annotation [40]. Overlapped modules were detected by using the codes adapted from WGCNA tutorials, and Fisher's exact test was used to calculate p-value for each of the pairwise overlaps.

For species comparison, syntenic orthologues [41] were used, with manual correction for a small number of C_4_ genes. For examples, selection of function orthologues from tandem repeated gene families (e.g., NADP-MDH and PPDK-RP) was adjusted by expression pattern similarity between species; missing orthologues (e.g., PEPC) due to lack of syntenies in rice were added based on a combination of sequence similarity and expression pattern.

### Differentially gene co-expression network analysis

Differentially co-expressed gene modules were identified by DiffCoEx with modification [42]. DiffCoEx was originally designed to cluster genes using a novel dissimilarity measurement computed from the topological overlap of the correlation changes between biological conditions. In this study, this method was adapted to detect gene correlation changes between species. Intuitively, it would detect genes that are significantly co-expressed in one species but not the other. The original code did not separate the positive and negative correlations. It was modified in this study to separate the two types of correlations by an extra step of clustering. The threshold of differentially co-expression module detection was increased, so that only genes with high contrast of connectivity between species were included. More specifically, the cutoff was set as difference of correlation greater than 0.7 in more than 10% gene pairs, and the modules with gene number less than 30 were discarded.

### Identification of C4 candidates

**Fig 1:**
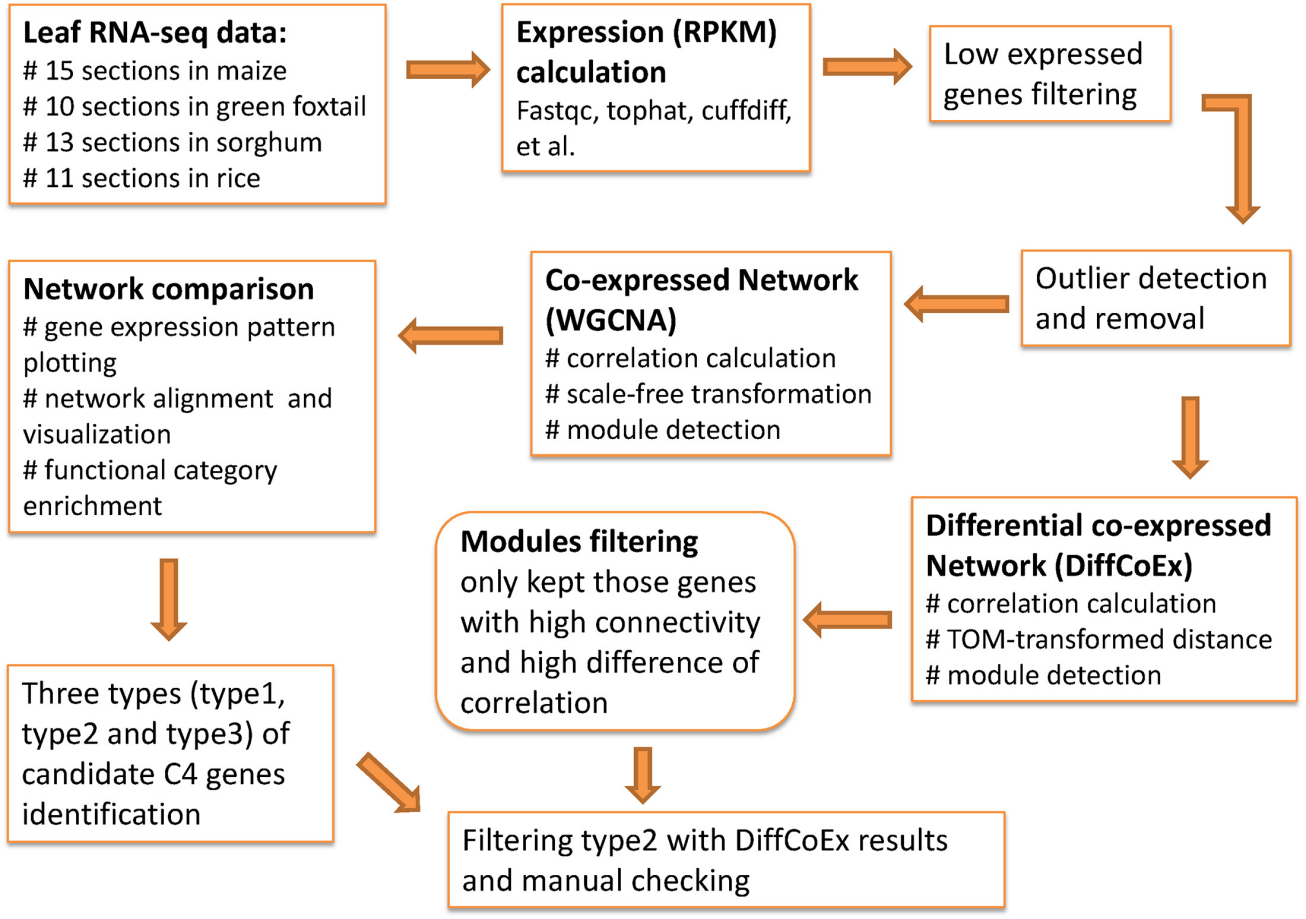
Workflow chart of this study.

C_4_ candidate genes were defined as those found in C_4_ modules (defined by co-expression modules which showed similar gene expression patterns as classic C_4_ genes, see below) of at least two C_4_ species in consideration of species specification/divergence, and then categorized into three sub-types by comparing with rice: (I) genes showed similar expression pattern as C_4_ modules but were lowly expressed in rice, e.g., the expression levels (mean of one third of all sections from the tip) were > = 1.5-fold lower in rice compared to C_4_ species; (II) genes showed different expression patterns in rice comparing with C_4_ species; and (III) genes whose syntenic orthologues were present in all three C_4_ species but absent in rice. We presume that genes should be differentially co-expressed once their expression patterns were changed between species, thus we then filtered the type II of C_4_ candidate genes with the DiffCoEx results, and only retained those that were differentially co-expressed in at least two out of three comparisons (maize vs. rice, green foxtail vs. rice, and sorghum vs. rice) with the same direction, either lower or higher correlated in C_4_ species than in rice. Finally, the expression patterns of C_4_ candidate genes were manually checked. The workflow chart of this study was showed in Fig 1.

## Results

### Gene co-expression network and modules comparison

**Fig 2:**
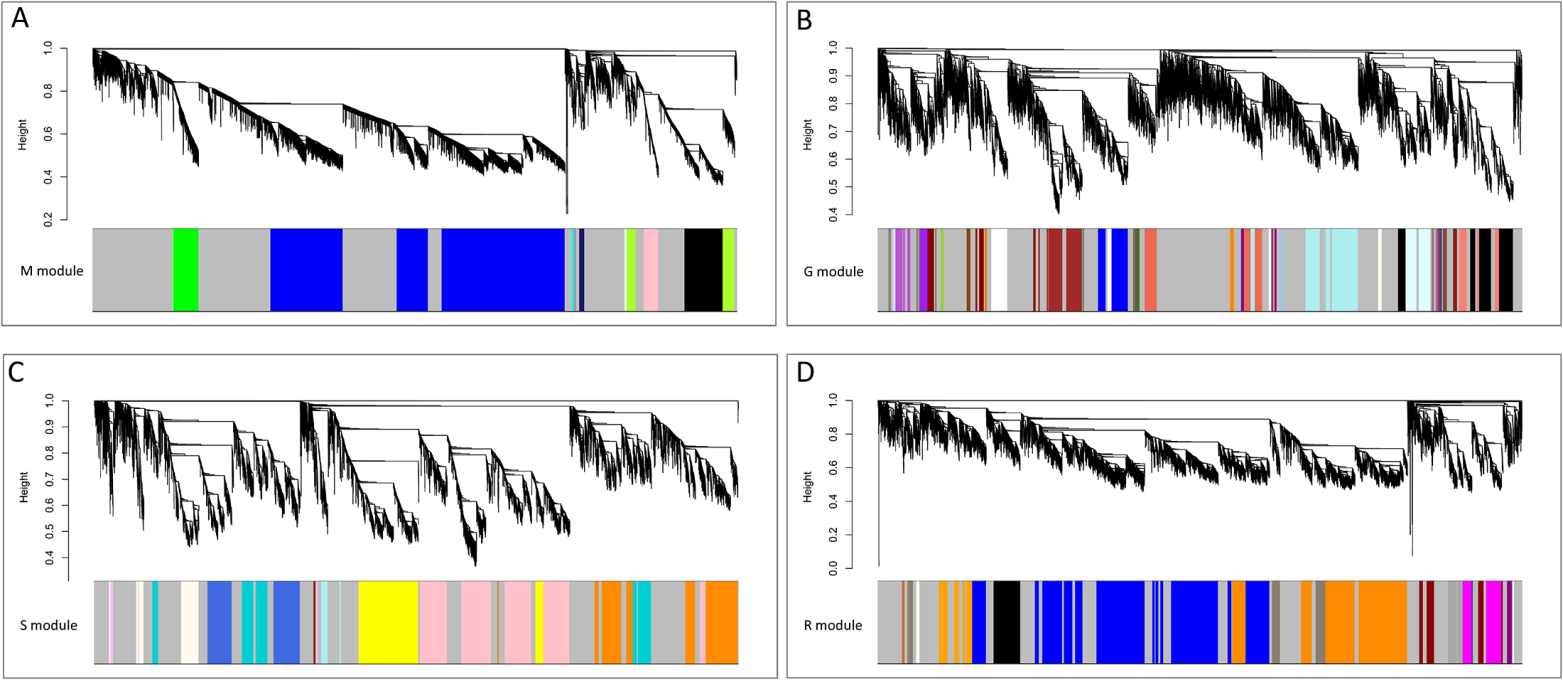
WGCNA co-expression network in maize (A), green foxtail (B), sorghum (C) and rice (D). Each line in the dendrogram represented an individual gene, and genes were assigned into modules with different colors in each species, respectively. Genes within grey module were unassigned genes that are not part of any module.

After removing the outlier (leaf section 1) and filtering out low expressed loci, genes of developing leaves from maize (18916 genes, 14 segments), green foxtail (17253 genes, 9 segments), sorghum (18119 genes, 12 segments) and rice (15964 genes, 11 sections), were used for co-expression network construction. Following the standard procedure of WGCNA, genes were assigned into different modules in each species according to their expression patterns along leaf gradients, and genes in the same module showed similar tendency due to a high Pearson correlation. Overall, we identified 11 modules in maize, 32 modules in green foxtail, 12 modules in sorghum and 14 modules in rice (Fig 2 and S2–S5 Figs), which account for 48%, 50%, 39% and 34% genes in each species, respectively.

We compared modules between species by following three criteria: gene expression patterns, overlapping orthologous genes, and overlapping enriched function categories based on MapMan annotation (S2 Table). We assumed that modules that show similar expression patterns and were enriched in the same function categories may have the same biological functions, and thus should have significantly overlapped orthologous gene pairs. On the other hand, cross-species modules with significantly overlapped orthologous gene pairs do not always have similar expression patterns and enriched functional categories, and may thus carry out different biological functions among species.

**Fig 3:**
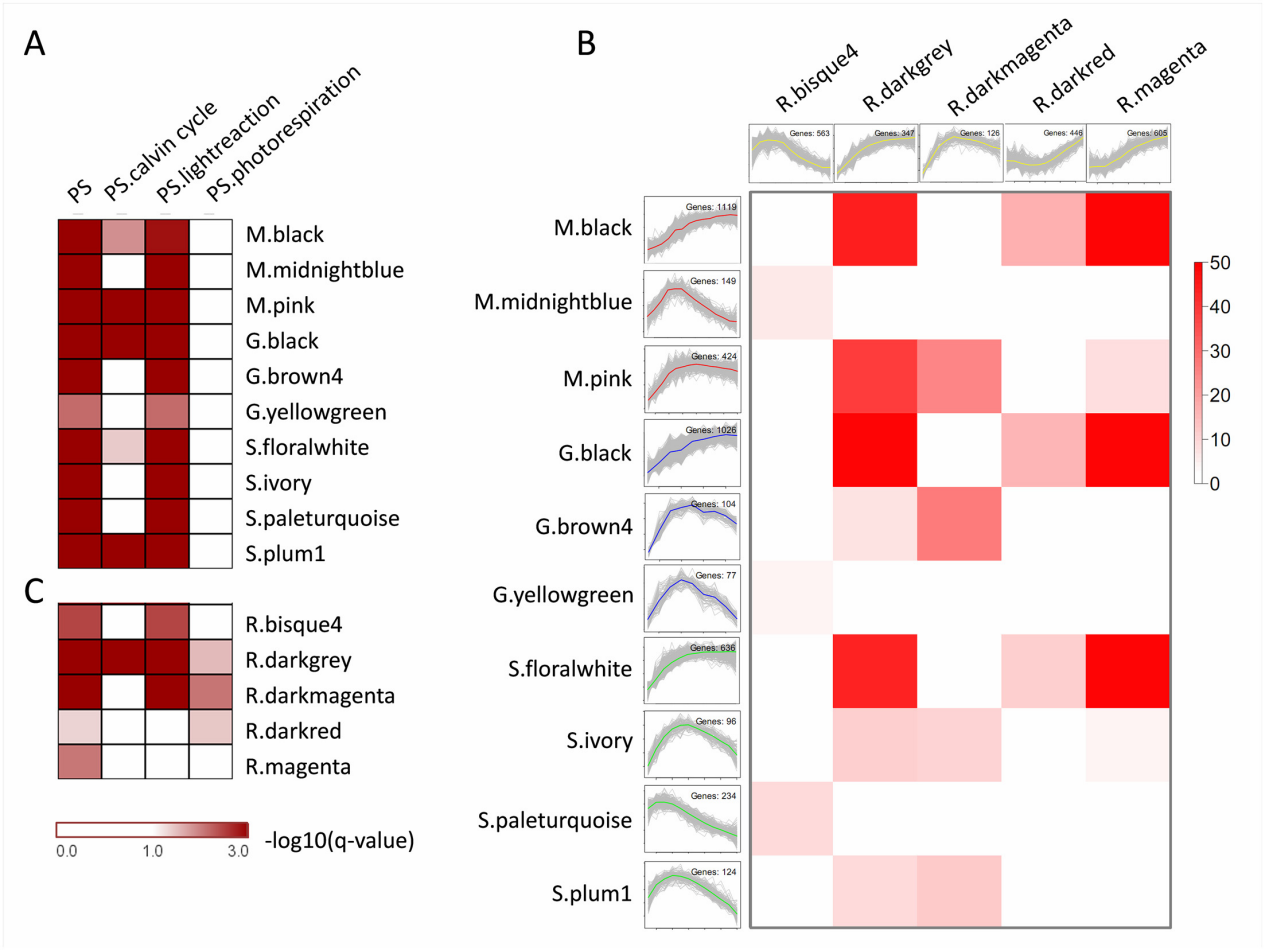
Photosynthesis enriched modules and their expression patterns in C_3_ and C_4_ species. A. PS modules enriched in C_4_ species; B. in C_3_ rice. C. Expression patterns and overlap conditions of PS modules. Modules of each species were prefixed with a capital letter respectively: maize (M), green foxtail (G), sorghum (S) and rice (R). Coloring of the modules overlap table encodes -log(p), with p being a Fisher's exact test p-value for the overlap of the two modules.

In this study, we aimed to discover genes that were involved in C_4_ photosynthesis, and thus focused mainly on the photosynthesis (PS) enriched modules in C_4_ species, specifically, M.black, M.pink and M.midnightblue in maize, S.floralwhite, S.ivory, S.paleturquoise and S.plum1 in soghurm, G.black, G.brown4 and G.yellowgreen in green foxtail (Fig 3A and S2 Table). We found that they were significantly overlapped in orthologous gene pairs (S6–S8 Figs). To examine the expression patterns of rice orthologues of those genes in PS enriched modules from C_4_ species, pairwise module comparison was performed between C_4_ species and rice (S9–S11 Figs). Interestingly, many rice modules (e.g., R.bisque4, R.darkgrey, R.darkmagenta, R.darkred and R.magenta) that showed significant overlaps with modules of C_4_ species (Fig 3B), were also enriched in PS related pathways (Fig 3C). Among them, R.darkgrey, overlapped with M.black in maize, S.floralwhite in sorghum and G.black in green foxtail, and showed similar expression patterns (Fig 3B and S9–S11 Figs), suggesting that the genes in these modules may be functionally important for photosynthesis and thus conserved among all four species. However, the expression patterns of these overlapping modules between rice and C_4_ species were different in some cases, e.g., genes in R.magenta and R.darkred, which significantly overlapped with M.black, S.floralwhite and G.black but showed different expression patterns (Fig 3B and S9–S11 Figs), may have changed their function as the species diverged.

It is worth noting that, in these PS enriched modules, three (R.darkgrey, R.darkmagenta and R.darkred) were photorespiration enriched in rice, while none in C_4_ species (Fig 3A and 3C), suggesting that photorespiration genes were co-expressed in rice but they were not co-expressed in C_4_ species.

### Identification of C_4_ modules based on classical C_4_ genes

**Fig 4:**
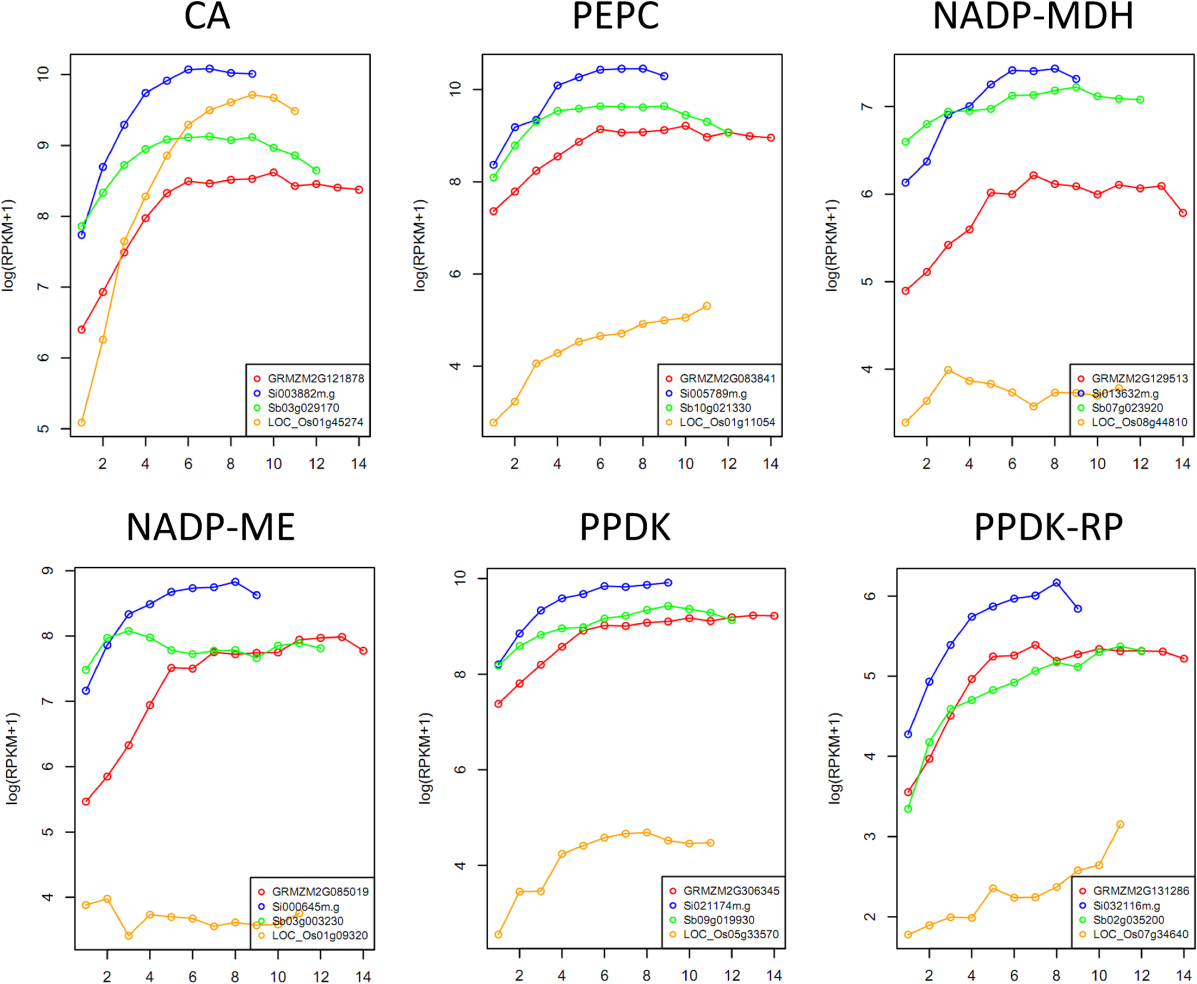
Expression pattern of classical C4 genes in four species. Gene IDs were plotted with different colors, e.g., red, blue, green and yellow for maize, green foxtail, sorghum and rice, respectively. CA: carbonic anhydrase, PEPC: phosphoenolpyruvate carboxylase, NADP-MDH: NADP-malate dehydrogenase, NADP-ME: NADP-malic enzyme, PPDK: pyruvate orthophosphate dikinase, and PPDK-RP: PPDK regulatory protein.

We noticed that classical C_4_ genes, e.g., carbonic anhydrase (CA), phosphoenolpyruvate carboxylase (PEPC), NADP-malate dehydrogenase (NADP-MDH), NADP-malic enzyme (NADP-ME), pyruvate orthophosphate dikinase (PPDK) and PPDK regulatory protein (PPDK-RP), were grouped in the same module that was enriched in PS related genes in maize (M.pink) and green foxtail (G.black), and had an increasing expression profile from the base to tip along the leaf (Fig 4). In addition, in sorghum, four of them were found in S.floralwhite, and two (NADP-MDH and NADP-ME) in S.grey. The separation of NADP-MDH and NADP-ME from S.floralwhite to grey (genes that are not clustered into modules) may be due to the fact that both of them have tandem duplicated paralogs in the genome (e.g., Sb07g023910 vs. Sb07g023920 and Sb03g003220 vs. Sb03g003230). Based on these observations, we assumed that genes in PS modules, e.g. M.black and M.pink in maize, G.black and G.brown4 in green foxtail, S.floralwhite in sorghum, which contained and showed similar expression patterns of classical C_4_ genes, contained C_4_-related candidate genes, and will thus be referred to as “C_4_ modules” hereafter. In rice, orthologues of CA, PEPC and PPDK showed an expression pattern in R.darkgrey that was similar to the C_4_ modules. Although CA showed a similar expression level in both C_3_ and C_4_ species, the expression of PEPC and PPDK was much lower in rice than in the other three C_4_ species (Fig 4). The remaining three rice orthologues of classical C_4_ genes (NADP-MDH, NADP-ME and PPDK-RP), which were grouped in the R.grey module, had a much lower expression level, and showed different expression pattern compared with their C_4_ orthologues. These results suggest that classical C_4_ genes either decreased in their expression level or changed their expression pattern in rice. Thus, the characteristics of classical C_4_ genes can be used as a criterion to define other C_4_-related candidate genes (see Materials and Methods). Three major categories of C_4_ genes were characterized: similar expression pattern with higher expression in C_4_ than in C_3_ species (type I); different expression patterns in rice compared to C_4_ species (type II), and syntenic orthologues present in all three C_4_ species but absent in rice (type III). Based on these criteria and the expression pattern examination of syntenic orthologues in overlapping modules between C_3_ and C_4_ species, 478 genes, including 25 type I, 417 type II and 36 type III were identified as C_4_ candidate genes. We further inspected the type III genes using SynFind in CoGe (https://genomevolution.org/CoGe/SynMap.pl), and found that six of these genes had “no syntenic regions” in the rice genome, while seven of them had "hits" within the rice syntenic regions, but the “hits” were not annotated as genes. These 13 genes were excluded because of the difficulty in verifying whether they were indeed present or absent in rice. The remaining 23 type III genes, which are positioned within all three C_4_ genomic regions that were in synteny with rice and had no rice homologs detected in these regions, were kept (S3 Table).

### Differential gene co-expression network

To identify genes that had differential co-expression network patterns in rice compared with C_4_ species, we use DiffCoEx, an algorithm that divides genes into different modules by calculating the correlation difference between rice and C_4_ species. This method allows the detection of orthologous modules in which genes show high correlation in one species, but low or no correlation in another species (S12–S14 Figs). In total 4249, 2965 and 7517 genes, which were clustered into 12, 11 and 18 modules respectively, were identified as differentially co-expressed by comparing rice with the other three C_4_ species (maize, sorghum and green foxtail) (S15–S17 Figs).

**Fig 5:**
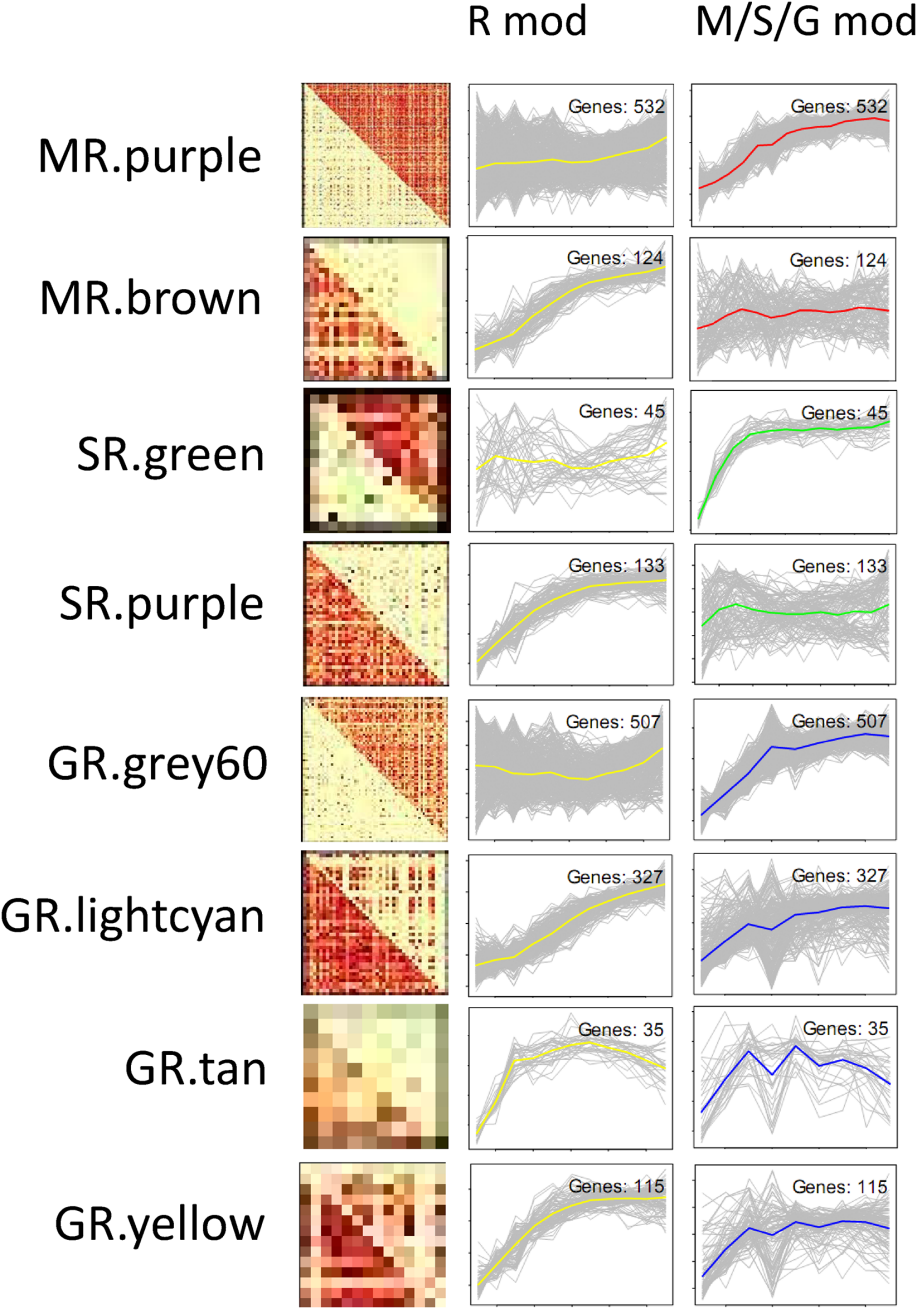
Differential co-expressed photosynthesis enriched modules between rice (R) and C_4_ species of maize (M), green foxtail (G) and sorghum (S). The first column represents heatmaps (the red color indicates positive correlations and blue indicates negative) of the modules, the second column (R Mod) indicated the corresponding gene expression profiles in rice; and the third column (M/S/G Mod) represented corresponding gene expression profiles in maize (red line), sorghum (green line) and green foxtail (blue line).

Here, we also focused on eight modules whose expression patterns were similar to the C_4_ modules, e.g. the gene expression was increased from base to tip, similar to PS genes (MR.purple and MR.brown; SR.green and SR.purple; GR.grey60, GR.lightcyan, GR.tan and GR.yellow) were identified (Fig 5). Of which, MR.purple, SR.green and GR.grey60 were more correlated, while the remaining five were less correlated, in C_4_ species than in rice. As expected, most of them were significantly enriched in PS related genes (S4 Table). To identify additional genes that may be important for C_4_ photosynthesis, we selected 155 genes that showed increased correlation in at least two out of three C_4_ species and 172 genes that showed decreased correlation in at least two C_4_ species compared to rice.

### Identification of C_4_ candidate genes

We filtered 124 genes from the type II C_4_ candidates identified via DiffCoEx, assuming that genes that had different expression pattern between rice and C_4_ species in the co-expression network (WGCNA), should be differentially co-expressed. After manually inspecting the expression patterns between C_3_ and C_4_ species, 128 C_4_ candidate genes (S5 Table), including 25 type I, 80 type II and 23 type III, were identified. As expected, these included many classical C_4_ genes. For example, PEPC and PPDK were identified as type I, and NADP-ME, NADP-MDH and PPDK-RP were identified as type II C_4_ genes (S5 Table). In addition, other well-known important C_4_ genes, such as aspartate aminotransferase (AST, GRMZM5G836910, type II) were also identified. The expression level of AST increased from base to tip in three C_4_ species, but, the expression trend of its rice orthologue (grouped in R.blue) was reversed (S18 Fig). In summary, the majority (63%) of C_4_ candidate genes had different expression patterns between C_4_ and rice, while a smaller proportion had either an elevated (20%) or a novel (18%) expression pattern or in C_4_ plants, possibly to accommodate the evolutionary transition from C_3_ to C_4_ photosynthesis in these species.

**Fig 6:**
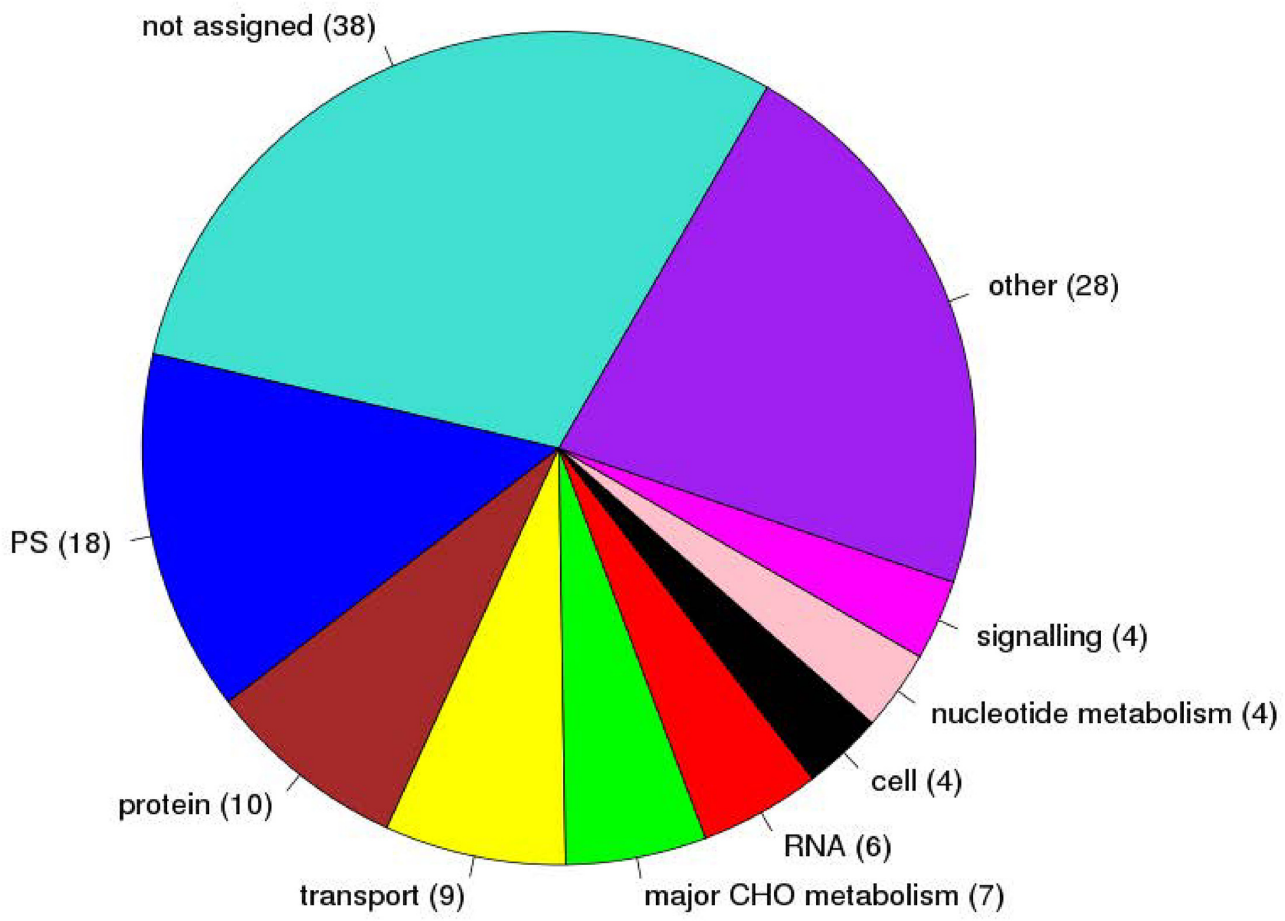
Functional distribution of 128 C_4_ candidate genes. Categories with gene number less than four were assigned into other.

Based on the MapMan category annotation, 70% (90/128) of the genes were assigned to known biological processes or pathways. The three most abundant functional groups, excluding genes classified as “not assigned”, were photosynthesis (PS) (18 genes), protein metabolism (10 genes) and transport (9 genes), followed by major carbohydrate (CHO) metabolism (7 genes) and RNA regulation (6 genes) (Fig 6). Among these 128 C_4_ candidate genes: 45% (57/128) were differentially expressed between BS and ME cells in both maize and green foxtail [22, 24], and another 49% (63/128) were identified as enriched in one cell type [18, 19, 22, 24] (S5 Table). The fact that the majority (94%, 120/128) of our C_4_ candidate genes were either BS- or ME-enriched, highly suggests that these C_4_ genes may play an important roles in C_4_ metabolism. In addition, by comparing with Wang et al. [21], 50% (64/128) of these C_4_ candidates were differentially expressed between maize foliar leaf blade (Kranz) and husk leaf sheath (non-Kranz), and 89% (57/64) of them were significant highly expressed in foliar expanded (FE) leaf, not the leaf primodia (S5 Table), indicated these candidates were mainly involved in C_4_ photosynthesis in leaves. Moreover, 81% (104/128) of these C_4_ candidates homologous were found to be differentially expressed in leaf gradients of Cleome gynandra [43], a NAD-ME type C_4_ dicot in an independent C_4_ lineages, and 45% (57/128) of them were found to show similar expression patterns between C. gynandra and maize (S5 Table). These may indicate the conservation role of these genes in C_4_ evolution. In addition to the well-known classic C_4_ genes, we also identified a set of genes, involved in carbohydrate metabolism, that seem to play a role in C_4_ photosynthesis. For example, GRMZM2G070605 and GRMZM2G066413, two triosephosphate phosphate translocators that transport Calvin cycle derived triosephosphates from the stroma to the cytosol for use in sucrose synthesis and other biosynthetic processes [44], and FBA, FBP and SBP, important enzymes controlling the metabolite flux in the Calvin cycle. Interestingly, six genes related to starch degradation were identified as C_4_ candidates, e.g. phosphoglucan phosphatase (SEX4, GRMZM2G052546), whose mutation partially blocked the starch degradation process and then influence the plant growth in Arabidopsis [45], and beta-amylases (GRMZM2G082034, GRMZM2G007939, GRMZM2G035749 and GRMZM2G347708), which play a central role in the complete degradation of starch to maltose [46]. Except for starch degradation related genes, one gene (GRMZM2G121612), responsible for starch biosynthesis, was identified. We also identified several sugar transporters that may take part in C_4_ photosynthesis. For example, SUT1/2 (GRMZM2G087901 and GRMZM2G034302) and STP1 (GRMZM5G801949), which are crucial for efficient phloem loading of sucrose in maize leaves [47].

In addition, we identified eight transcription factors that might participate in C_4_ photosynthesis (S5 Table), including MYBs, ARFs, and G2-like TF (GRMZM2G052544). GRMZM2G052544 is a homolog of APL (ALTERED PHLOEM DEVELOPMENT), which is involved in promoting phloem differentiation and repressing xylem differentiation during vascular development in Arabidopsis [48]. GRMZM2G052544 and its syntenic orthologue (Si017608m.g) were BS-enriched (S5 Table), showed high expression in expanded leaf in C_4_ species and low expression in rice, which indicated it may be essential for C_4_ photosynthesis.

According to Gene Ontology (GO), 23 type III C_4_ candidates, involved in many biological processes such as photosynthesis (GO:0015979), oxidation-reduction process (GO:0055114), cellular component such as membrane (GO:0016020), chloroplast (GO:0009507), chloroplast thylakoid membrane (GO:0009535), and plasma membrane (GO:0005886), and chloroplast envelope (GO:0009941), molecular function such as DNA binding (GO:0003677) and protein binding (GO:0005515) (S6 Table) were annotated. These genes may also play an important role in the evolution of C_4_.

## Discussion

C_4_ photosynthesis is a complex metabolic pathway that relies on tight collaboration of many enzymes. The identity of many of the genes required for the proper function of C_4_, as well as their regulatory mechanisms, however, remain elusive. In this study, we combined gene co-expression and gene differentially co-expression networks, to identify candidate genes that may be necessary for C_4_ photosynthesis. Unlike previous studies that focused on the differential expression between bundle sheath and mesophyll at the gene [19, 20, 22, 24] or protein [16–18] level, we focused on the comparison of gene co-expression and differential gene co-expression relationships along a developmental leaf gradient among multiple C_3_ and C_4_ species.

### Possible evolution of C_4_ candidate genes

C_4_ photosynthesis is thought to have evolved mainly through gene/genome duplication, and subsequent functional innovation of pre-existing genes in C_3_ species [49, 50]. Leaf gradient RNA-seq data from C_3_ and C_4_ species provide us with a good opportunity to dissect the possible evolution of C_4_ candidate genes by tracking changes of gene expression patterns [51, 52]. Overall, the expression levels and patterns of C_4_ genes were similar within the three C_4_ species differed in C_3_ rice, suggesting that long term adaptive selection may have encouraged the formation of C_4_ photosynthesis, (e.g., adaptation to high temperature and low CO_2_) [4, 53–55], as previously suggested using different algorithms [50].

Based on our results, we suggest three possible evolution scenarios for the recruitment of genes into the C_4_ pathway (1) Genes that have expression patterns similar to known and well characterized photosynthetic genes, could have increased their expression levels in C_4_ species (e.g., type I), because C_4_ photosynthesis requires light-regulated high expression of genes in leaves [9, 56]; (2) Genes that exhibit an expression patterns different from photosynthetic genes, may have altered their expression patterns to obtain a novel function for C_4_ photosynthesis (e.g., type II). This explanation is consistent with a recent study, which demonstrated that C_4_ expression patterns were not present in the C_3_ ancestors, but were acquired during the evolutionary transition from C_3_ to C_4_ photosynthesis [11]; (3) Genes that have syntenic orthologues in C_4_ species but were absent from C_3_ rice (e.g., type III), may represent genes that were newly formed in C4 species, after the divergence of the C_3_ and C_4_ lineages, and may thus participate in new biological functions that do not exist in C_3_ plants.

### New insights into C_4_ photosynthesis genes

Using our selection criteria, some characterized C_4_ genes, such as carbonic anhydrase (CA, GRMZM2G121878) and phosphoenolpyruvate carboxykinase (PEPCK) were eliminated from the candidate list. CA, the first enzyme of the C_4_ carbon shuttle, was proposed to be a necessary enzyme for C_4_ photosynthesis. However, our results showed that, both its expression pattern and expression level were very similar among the examined C_3_ and C_4_ species, suggesting, as have recently been shown [57], that CA may not be a rate limiting enzyme for photosynthesis in C_4_ species.

Maize has two PEPCK genes. The expression of GRMZM2G001696 (max RPKM = 2573) was higher than that of GRMZM5G870932 (max RPKM = 791). However, only one PEPCK gene was identified in sorghum, green foxtail and rice, and the expression level of PEPCK in these species (max RPKM 158 in sorghum, 28 in green foxtail and 34 in rice) was much lower than that in maize, although the peak of their expression was at the leaf tip, as expected from C_4_ genes. Moreover, the expression patterns of PEPCK in green foxtail (G.floralwhite) and rice (R.darkred) were quite similar, and very different from maize (M.black) and sorghum (S.floralwhite, S2–S5 Figs). These results suggest that green foxtail, like rice, may not have a PEPCK regulated decarboxylation reaction as maize or sorghum. Another possibility is the function of PEPCK was limited in the NADP-ME type C_4_ photosynthesis species examined in this study, consistent with Zhu et al. [58] that hypothesized that only the NAD-ME type and NADP-ME type should be considered as distinct C_4_ subtypes, with the PEPCK pathway serving only as a supplement.

### Identification of additional C_4_ genes

Engineering C_4_ photosynthesis into C_3_ crops requires a deep understanding of the essential components in the C_4_ pathways. Despite the progresses that have been made in recent years, with the functional characterization of many important C_4_ genes, the number of genes that are essential for establishing C_4_ metabolism in C_3_ crops is still unknown [6]. Comparative transcriptomics has extensively proved to be a useful approach to identify novel C_4_ candidate genes [19, 22, 24]. By comparing the gene expression pattern among C_3_ and C_4_ species, we identified 38 novel C_4_ candidates, which were previously classified as functional unknown or not-assigned by the MapMan annotation. The vast majority of these newly identified genes (94.7%) were differentially expressed between the BS and ME cells (S5 Table). These candidate genes were highly co-expressed with classical C_4_ genes, and may thus require further characterization to discover their exact function during C_4_ photosynthesis. Twenty six of these genes were annotated by GO, as involved in biological processes such as photosynthesis (GO:0015979), chlorophyll biosynthetic process (GO:0015995), maltose metabolic process (GO:0000023), and molecular functions such as catalytic activity (GO:0003824), hydrolase activity (GO:0016787), phosphotransferase activity (GO:0016776), and many cellular component ontology associated with chloroplasts (GO:0009507, GO:0009570, GO:0009534, GO:0009535) and membranes (GO:0016020, GO:0005886, GO:0042651) (S7 Table).

In addition, a set of carbon metabolism related genes, including FBA, FBP and SBP that control the carbon flux during the Calvin cycle; TPT that transport triosephosphate out of the chloroplast, as well as starch synthase that control starch biosynthesis, were identified as essential for C_4_ metabolism, and should thus be considered when engineering C_4_ photosynthesis into C_3_ crops.

Very little is known about genes that are associated with Kranz anatomy and metabolite transportation in C_4_ leaves [59, 60]. Our method identified two sucrose transporters (SUT1 and SUT2) and two triosephosphate phosphate translocators (TPT1 and TPT2), that were shown to have important roles in the C_4_ carbon shuttle [27, 44], as well as several transporters associated with K^+^ efflux, transmembrane transporter activity and others (S4 Table). Due to the low number of developmental stages in our dataset, however, we could not identify any Kranz anatomy associated genes. With the growing availability of high resolution tissue/cell specific data, our method will be very useful in identifying and characterizing additional C_4_ candidate genes, and assist in our efforts to engineer C_4_ traits into C_3_ crops, to improve yield and feed the growing population of the world.

## Supporting Information

S1 Fig Clustering of samples used in this study. (TIF)

S2 Fig Gene expression patterns of modules for maize, green foxtail, sorghum and rice, respectively. (TIF)

S3 Fig Gene expression patterns of modules for maize, green foxtail, sorghum and rice, respectively. (TIF)

S4 Fig Gene expression patterns of modules for maize, green foxtail, sorghum and rice, respectively. (TIF)

S5 Fig Gene expression patterns of modules for maize, green foxtail, sorghum and rice, respectively. (TIF)

S6 Fig Pairwise overlaps of modules among rice and three C_4_ species, respectively. Each row and column of the table corresponds to one module (labeled by color as well as text) from two species, respectively. Numbers in the table indicate overlapped gene counts in the intersection of corresponding modules. Coloring of the table encodes -log(p), with p being the Fisher's exact test p-value for the overlap of the two modules. The more significant the overlap, the stronger the red color is.
(TIF)

S7 Fig Pairwise overlaps of modules among rice and three C_4_ species, respectively. Each row and column of the table corresponds to one module (labeled by color as well as text) from two species, respectively. Numbers in the table indicate overlapped gene counts in the intersection of corresponding modules. Coloring of the table encodes -log(p), with p being the Fisher's exact test p-value for the overlap of the two modules. The more significant the overlap, the stronger the red color is.
(TIF)

S8 Fig Pairwise overlaps of modules among rice and three C_4_ species, respectively. Each row and column of the table corresponds to one module (labeled by color as well as text) from two species, respectively. Numbers in the table indicate overlapped gene counts in the intersection of corresponding modules. Coloring of the table encodes -log(p), with p being the Fisher's exact test p-value for the overlap of the two modules. The more significant the overlap, the stronger the red color is.
(TIF)

S9 Fig Pairwise overlaps of modules among rice and three C_4_ species, respectively. Each row and column of the table corresponds to one module (labeled by color as well as text) from two species, respectively. Numbers in the table indicate overlapped gene counts in the intersection of corresponding modules. Coloring of the table encodes -log(p), with p being the Fisher's exact test p-value for the overlap of the two modules. The more significant the overlap, the stronger the red color is.
(TIF)

S10 Fig Pairwise overlaps of modules among rice and three C_4_ species, respectively. Each row and column of the table corresponds to one module (labeled by color as well as text) from two species, respectively. Numbers in the table indicate overlapped gene counts in the intersection of corresponding modules. Coloring of the table encodes -log(p), with p being the Fisher's exact test p-value for the overlap of the two modules. The more significant the overlap, the stronger the red color is.
(TIF)

S11 Fig Pairwise overlaps of modules among rice and three C_4_ species, respectively. Each row and column of the table corresponds to one module (labeled by color as well as text) from two species, respectively. Numbers in the table indicate overlapped gene counts in the intersection of corresponding modules. Coloring of the table encodes -log(p), with p being the Fisher's exact test p-value for the overlap of the two modules. The more significant the overlap, the stronger the red color is.
(TIF)

S12 Fig Gene expression patterns of modules corresponding to S16–S18 Figs, respectively. (TIF)

S13 Fig Gene expression patterns of modules corresponding to S16–S18 Figs, respectively. (TIF)

S14 Fig Gene expression patterns of modules corresponding to S16–S18 Figs, respectively. (TIF)

S15 Fig Differential expressed modules detected between rice (R) and three C4 species of maize (M), sorghum (S) and green foxtail (G). (TIF)

S16 Fig Differential expressed modules detected between rice (R) and three C4 species of maize (M), sorghum (S) and green foxtail (G). (TIF)

S17 Fig Differential expressed modules detected between rice (R) and three C4 species of maize (M), sorghum (S) and green foxtail (G). (TIF)

S18 Fig Different expression pattern of aspartate aminotransferase (AST) between rice and three C_4_ species. (TIF)

S1 Table The comparison between qPCR and RNA-seq. (XLSX)

S2 Table Functional category enrichment of gene co-expressed modules (WGCNA) in four species. Numbers are—log_10_ transformed q values.
(XLSX)

S3 Table Inspection of type III candidate C_4_ genes based on SynFind in CoGe. (XLSX)

S4 Table Functional category enrichment of differential gene co-expressed modules (DiffCoEx) between rice and three C_4_ species. Numbers are—log10 transformed q values.
(XLSX)

S5 Table General information of 128 candidate C_4_ genes and comparison to other datasets. (XLSX)

S6 Table GO term of type III candidate C_4_ genes. (XLSX)

S7 Table GO term of 38 functional unknown candidate C_4_ genes. (XLSX)
