# Supplementary figures and images for: Identification of Photosynthesis-Associated C4 Candidate Genes through Comparative Leaf Gradient Transcriptome in Multiple Lineages of C3 and C4 Species
Source: PLoS One. 2015 Oct 14;10(10):e0140629. doi: 10.1371/journal.pone.0140629 (PMC4605685; doi:10.1371/journal.pone.0140629)

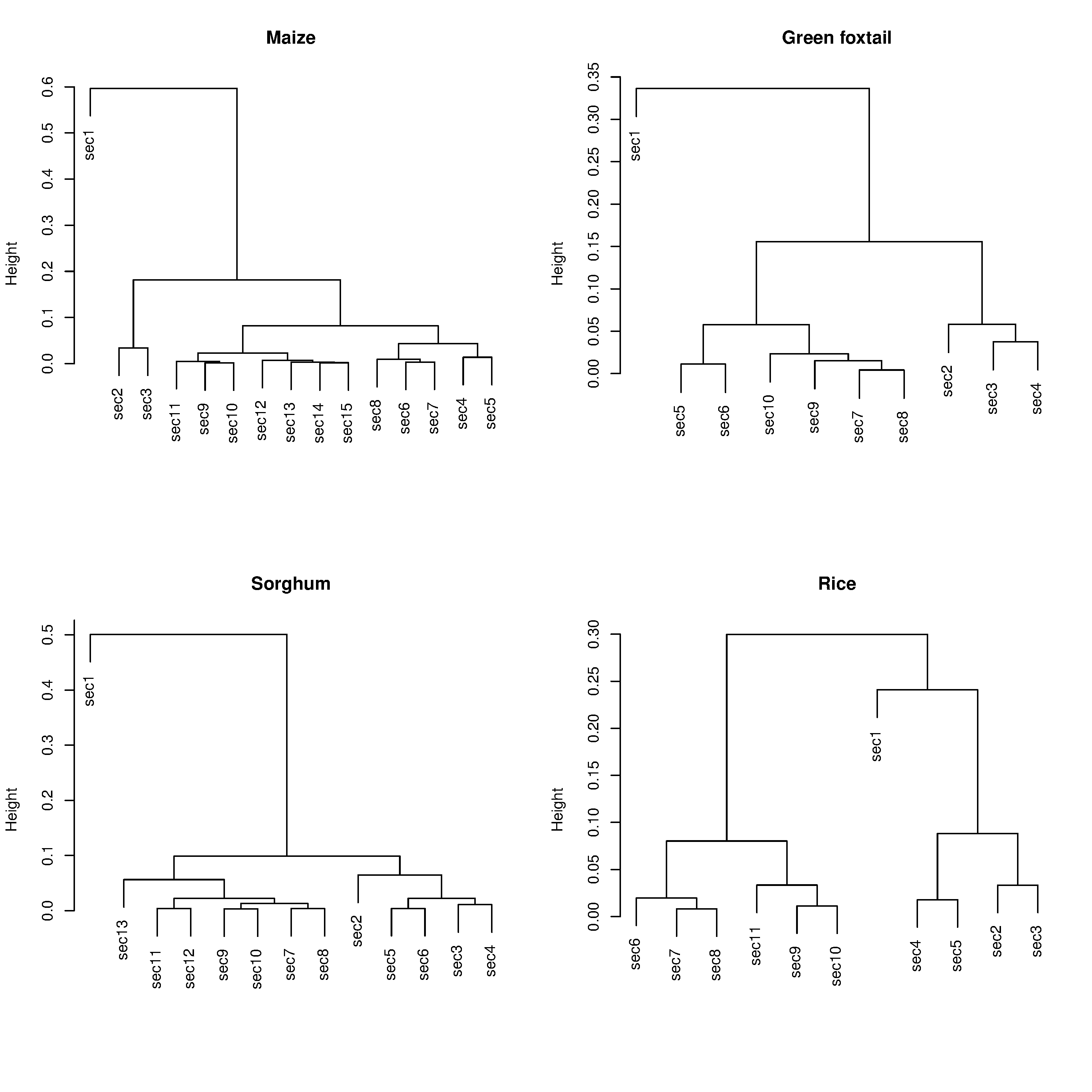

Supplement: S1 Fig — (TIF) [file pone.0140629.s001.tif]

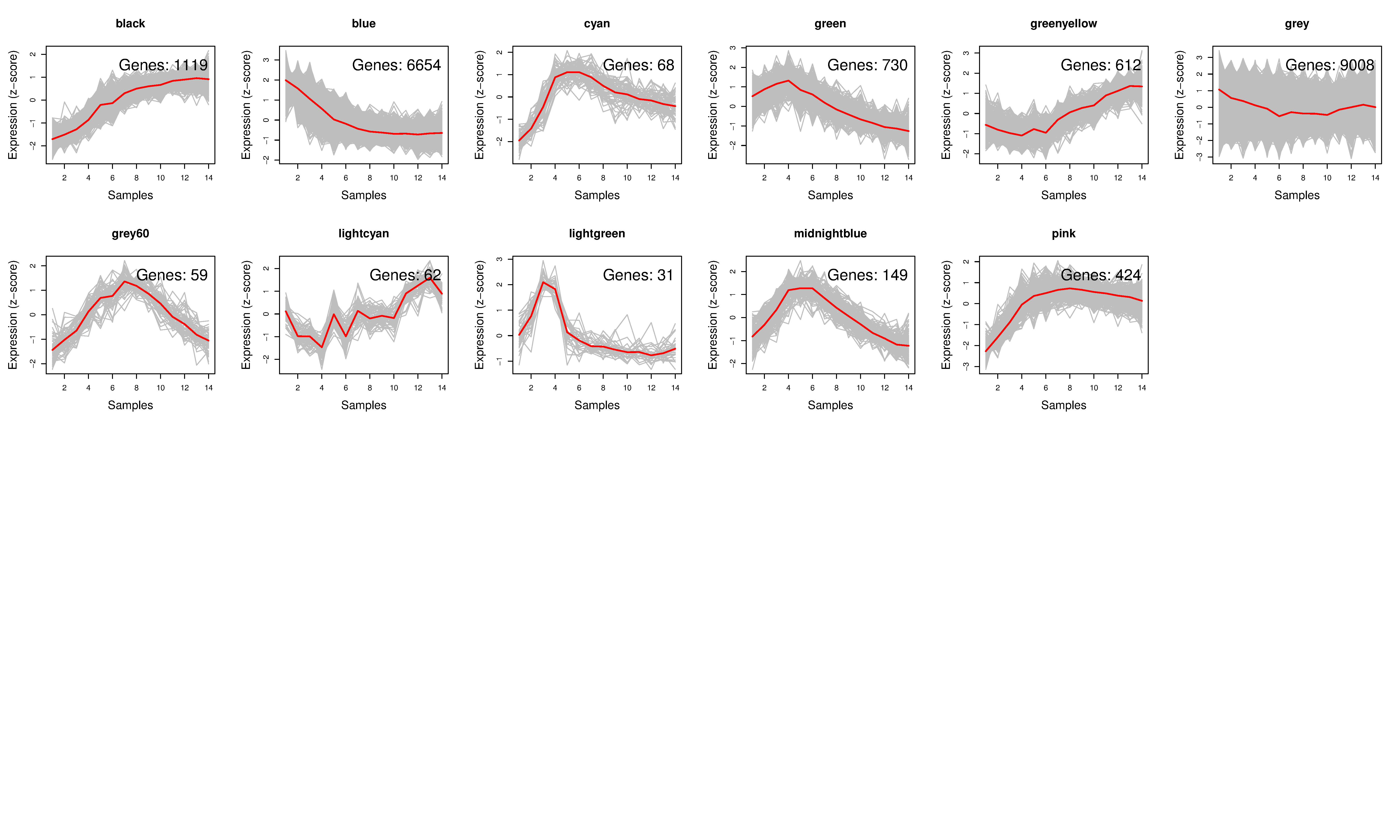

Supplement: S2 Fig — (TIF) [file pone.0140629.s002.tif]

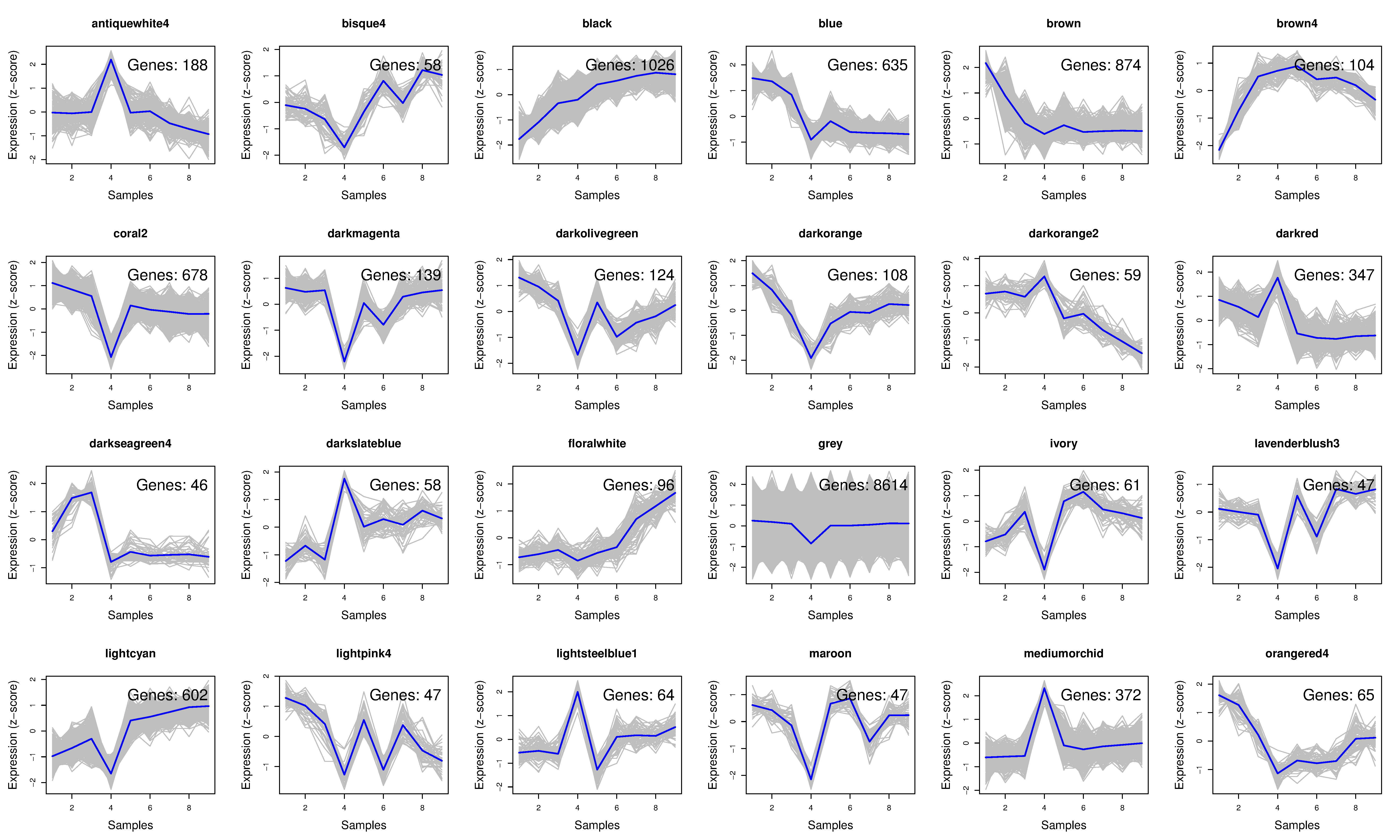

Supplement: S3 Fig — (TIF) [file pone.0140629.s003.tif]

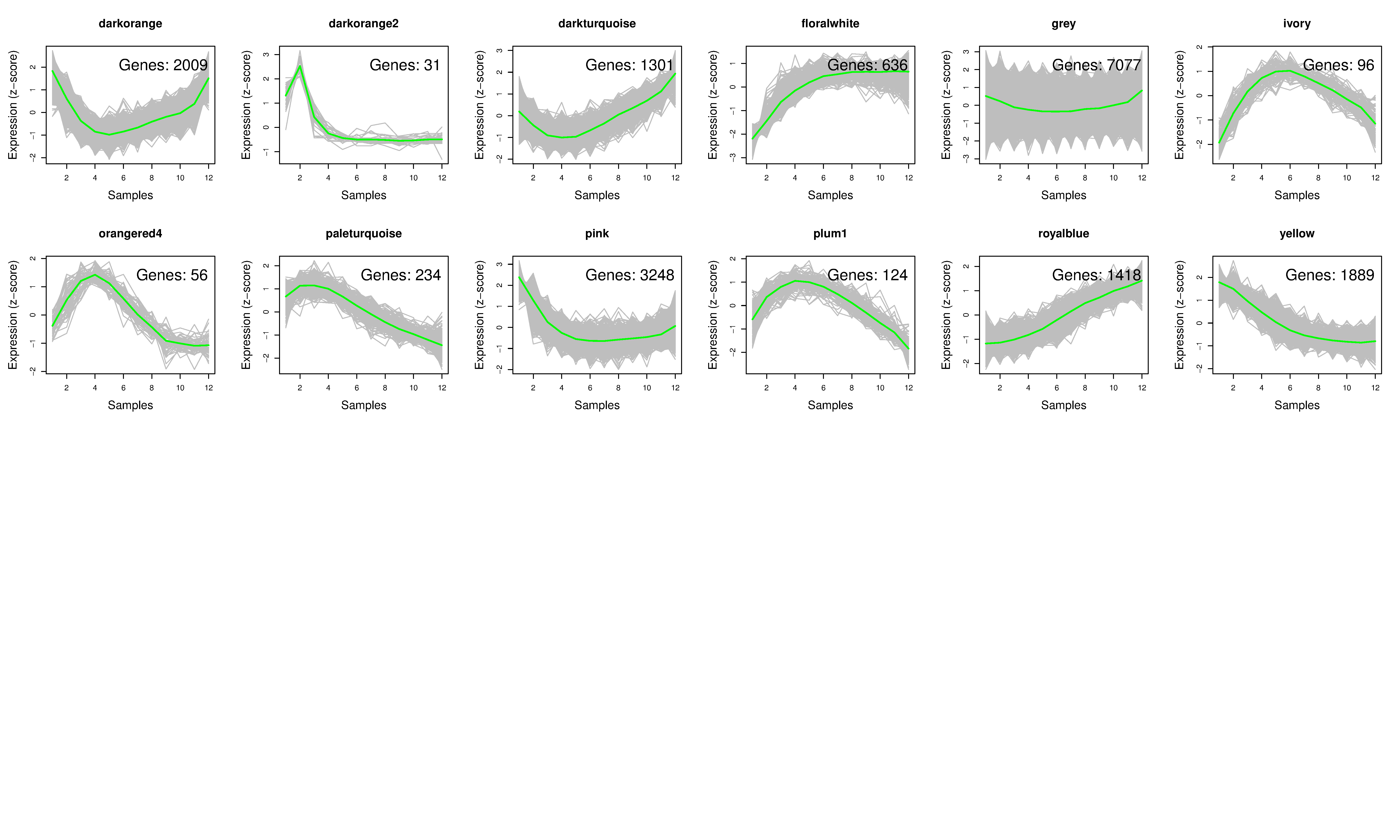

Supplement: S4 Fig — (TIF) [file pone.0140629.s004.tif]

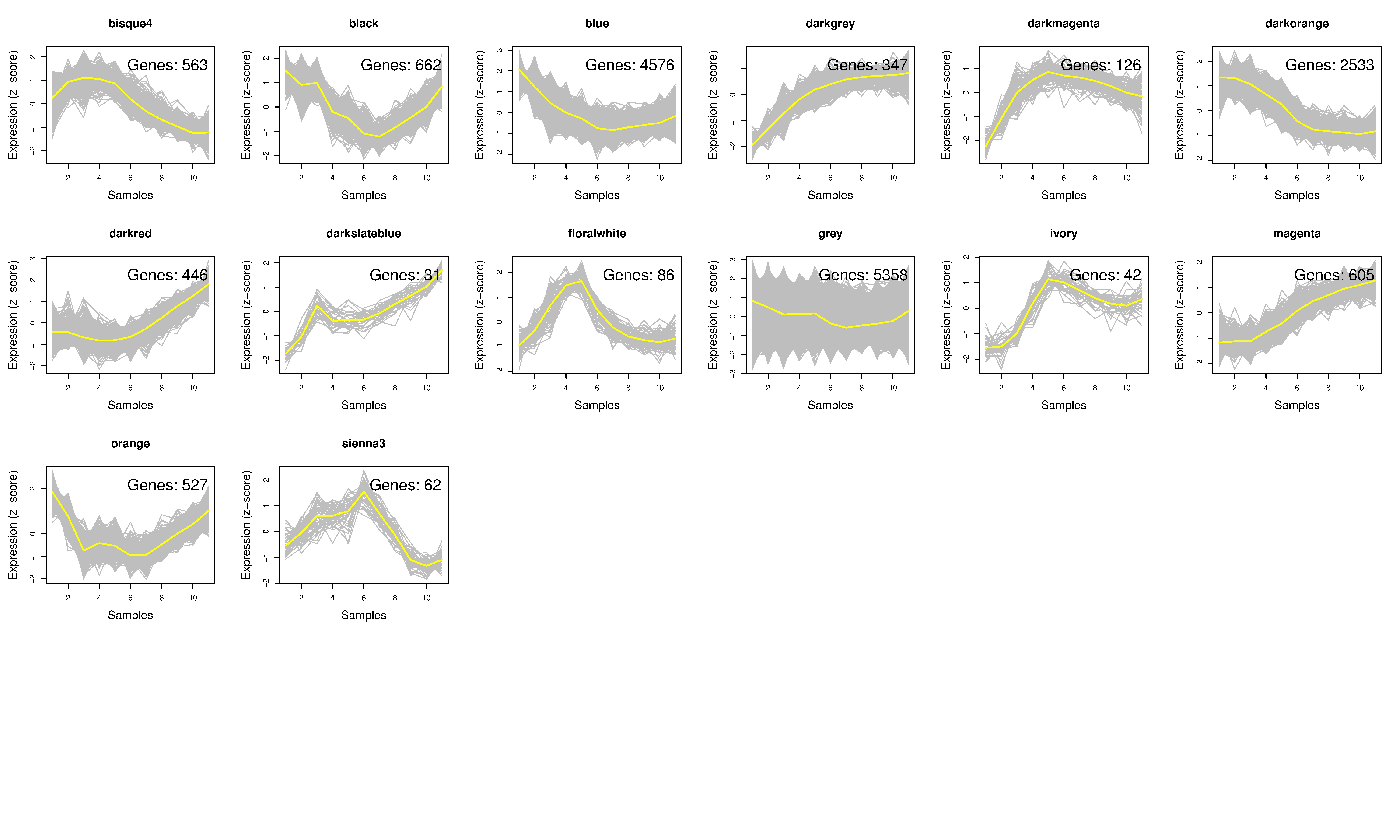

Supplement: S5 Fig — (TIF) [file pone.0140629.s005.tif]

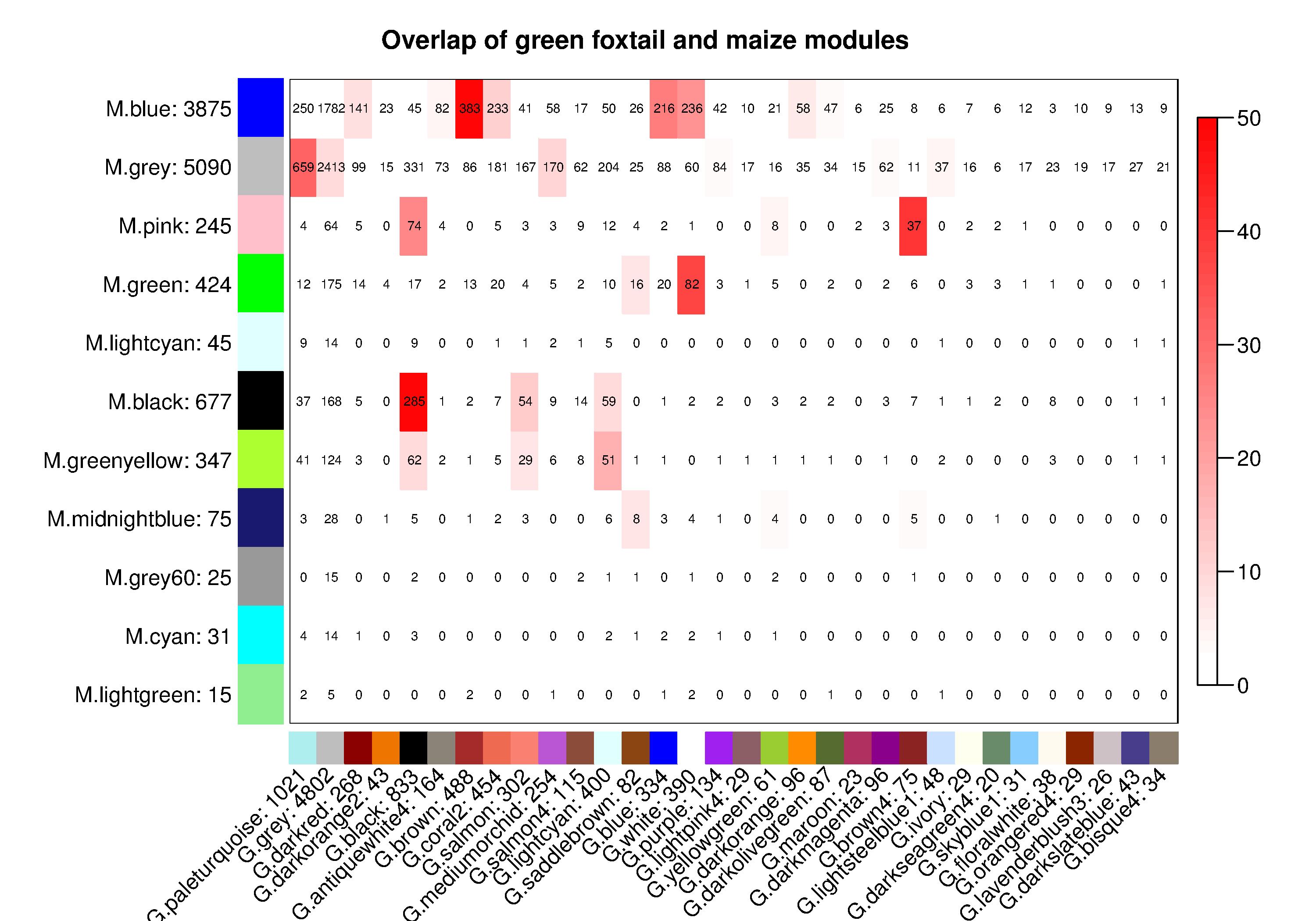

Supplement: S6 Fig — Each row and column of the table corresponds to one module (labeled by color as well as text) from two species, respectively. Numbers in the table indicate overlapped gene counts in the intersection of corresponding modules. Coloring of the table encodes -log(p), with p being the Fisher's exact test p-value for the overlap of the two modules. The more significant the overlap, the stronger the red color is. (TIF) [file pone.0140629.s006.tif]

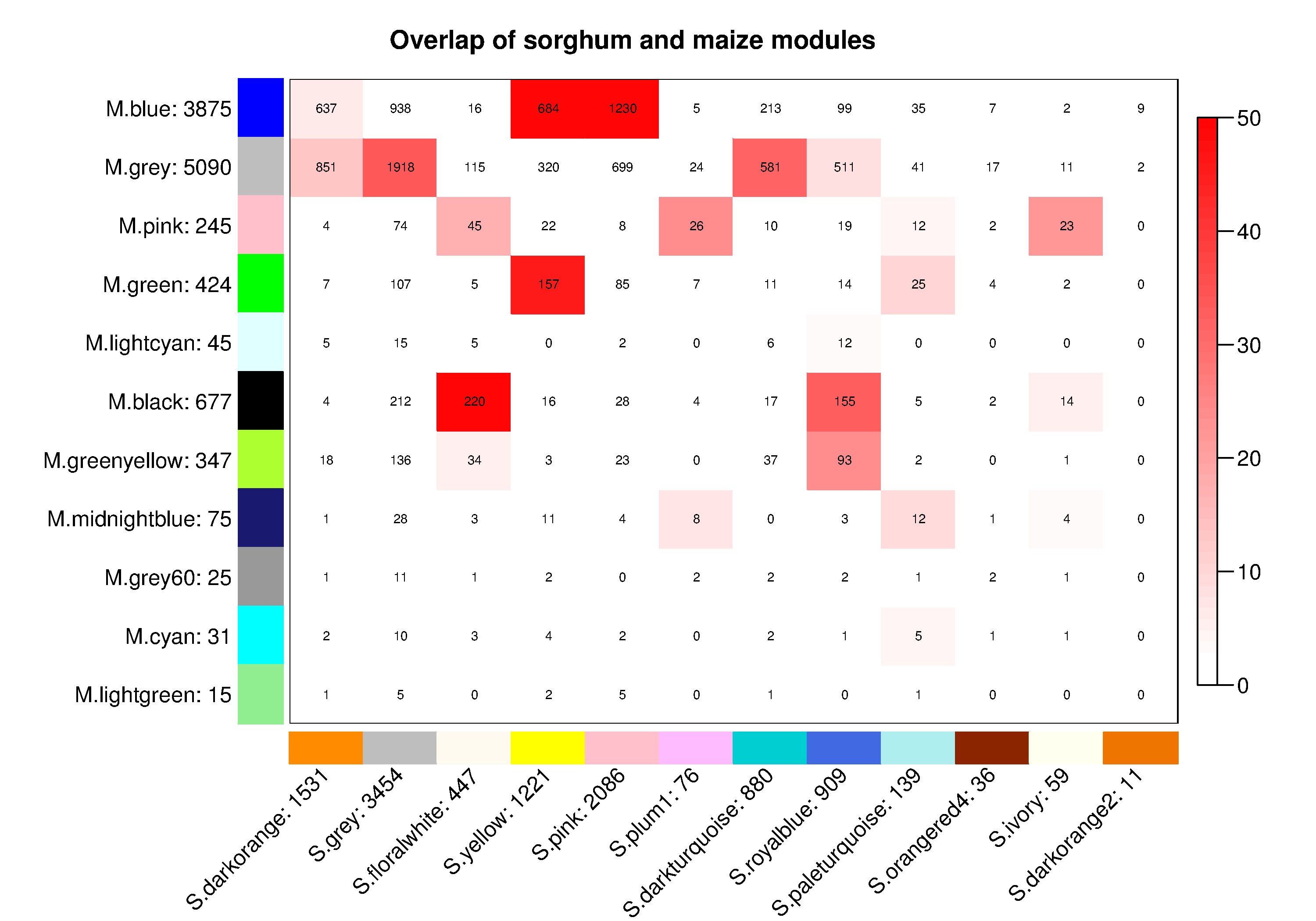

Supplement: S7 Fig — Each row and column of the table corresponds to one module (labeled by color as well as text) from two species, respectively. Numbers in the table indicate overlapped gene counts in the intersection of corresponding modules. Coloring of the table encodes -log(p), with p being the Fisher's exact test p-value for the overlap of the two modules. The more significant the overlap, the stronger the red color is. (TIF) [file pone.0140629.s007.tif]

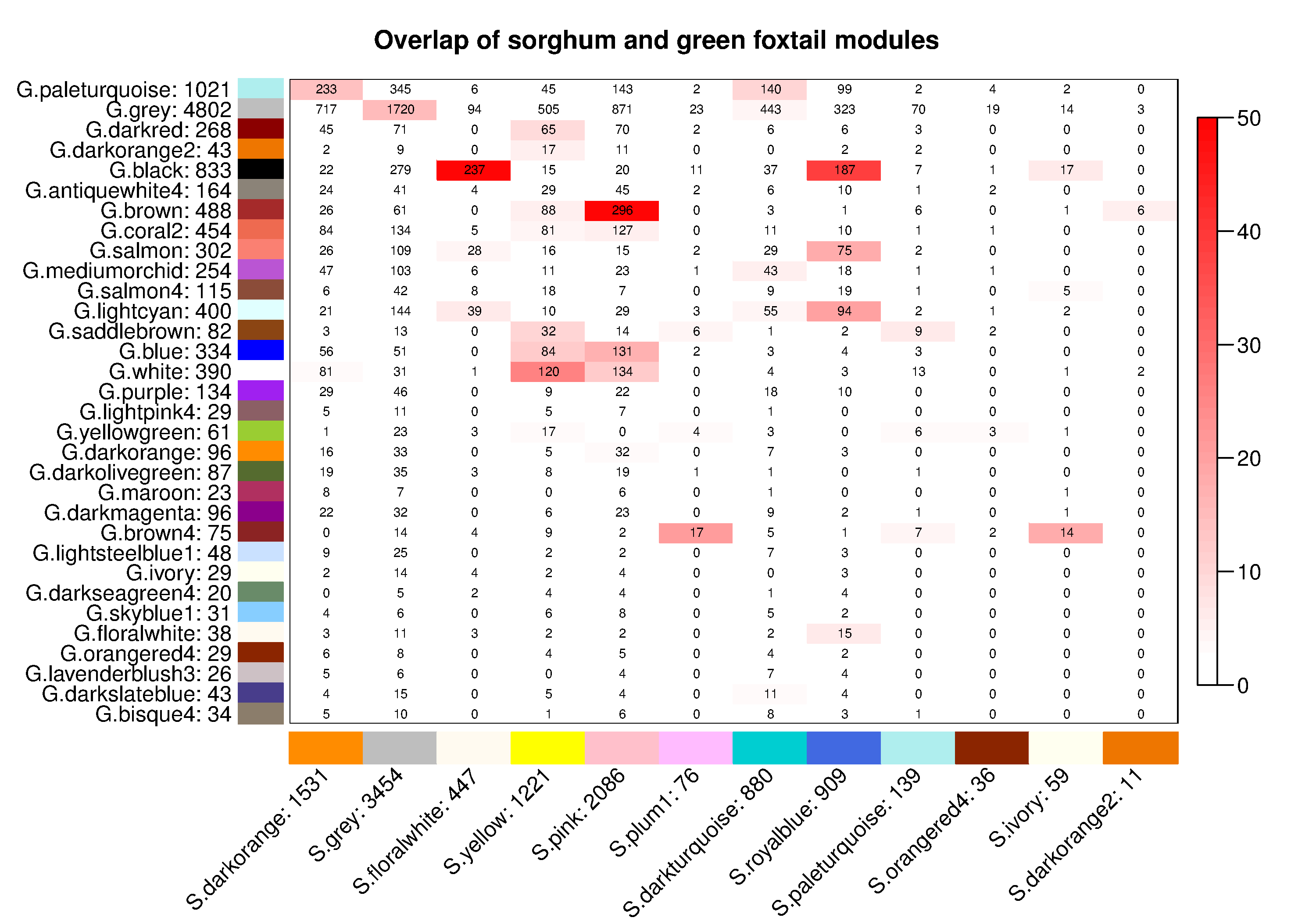

Supplement: S8 Fig — Each row and column of the table corresponds to one module (labeled by color as well as text) from two species, respectively. Numbers in the table indicate overlapped gene counts in the intersection of corresponding modules. Coloring of the table encodes -log(p), with p being the Fisher's exact test p-value for the overlap of the two modules. The more significant the overlap, the stronger the red color is. (TIF) [file pone.0140629.s008.tif]

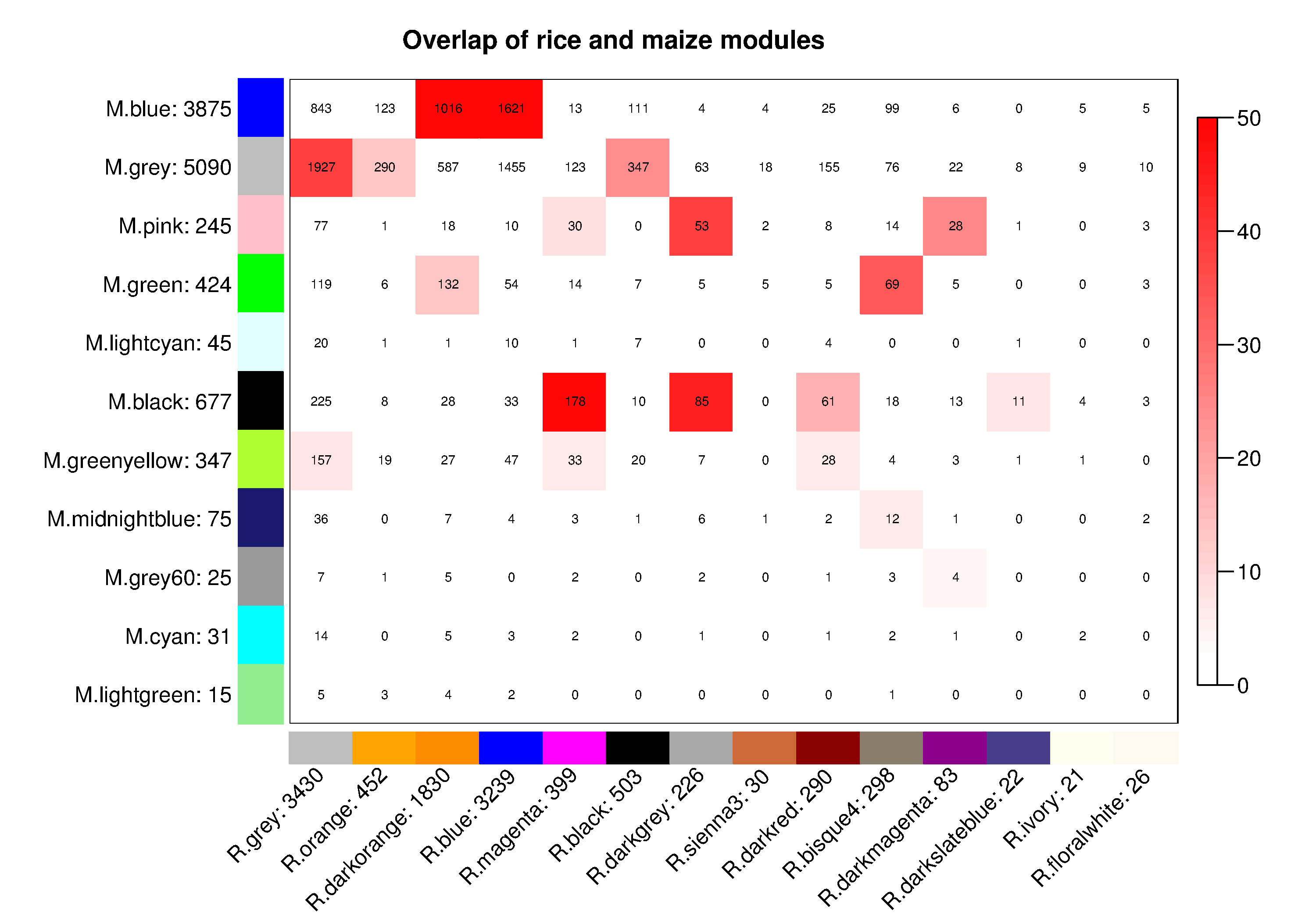

Supplement: S9 Fig — Each row and column of the table corresponds to one module (labeled by color as well as text) from two species, respectively. Numbers in the table indicate overlapped gene counts in the intersection of corresponding modules. Coloring of the table encodes -log(p), with p being the Fisher's exact test p-value for the overlap of the two modules. The more significant the overlap, the stronger the red color is. (TIF) [file pone.0140629.s009.tif]

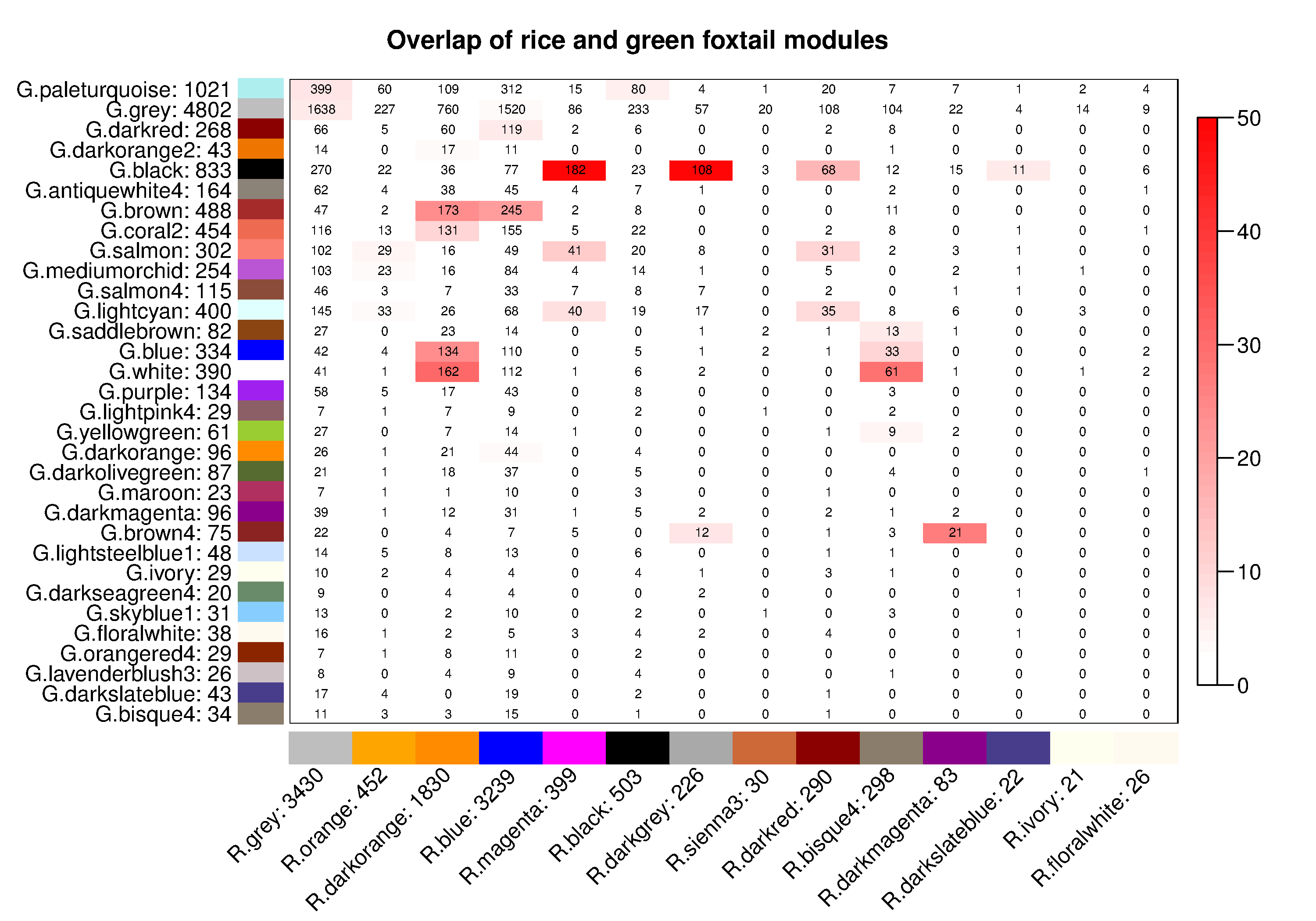

Supplement: S10 Fig — Each row and column of the table corresponds to one module (labeled by color as well as text) from two species, respectively. Numbers in the table indicate overlapped gene counts in the intersection of corresponding modules. Coloring of the table encodes -log(p), with p being the Fisher's exact test p-value for the overlap of the two modules. The more significant the overlap, the stronger the red color is. (TIF) [file pone.0140629.s010.tif]

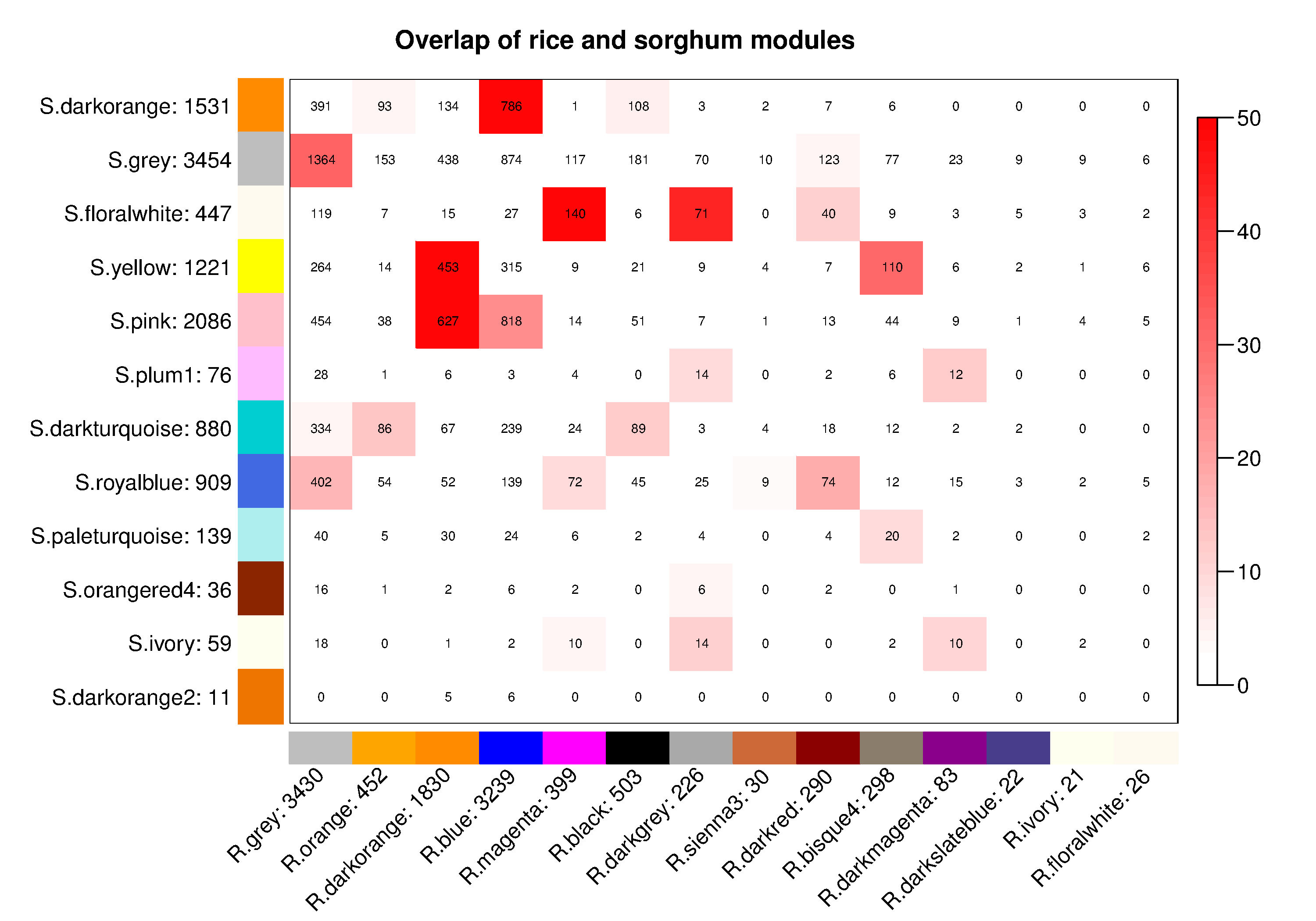

Supplement: S11 Fig — Each row and column of the table corresponds to one module (labeled by color as well as text) from two species, respectively. Numbers in the table indicate overlapped gene counts in the intersection of corresponding modules. Coloring of the table encodes -log(p), with p being the Fisher's exact test p-value for the overlap of the two modules. The more significant the overlap, the stronger the red color is. (TIF) [file pone.0140629.s011.tif]

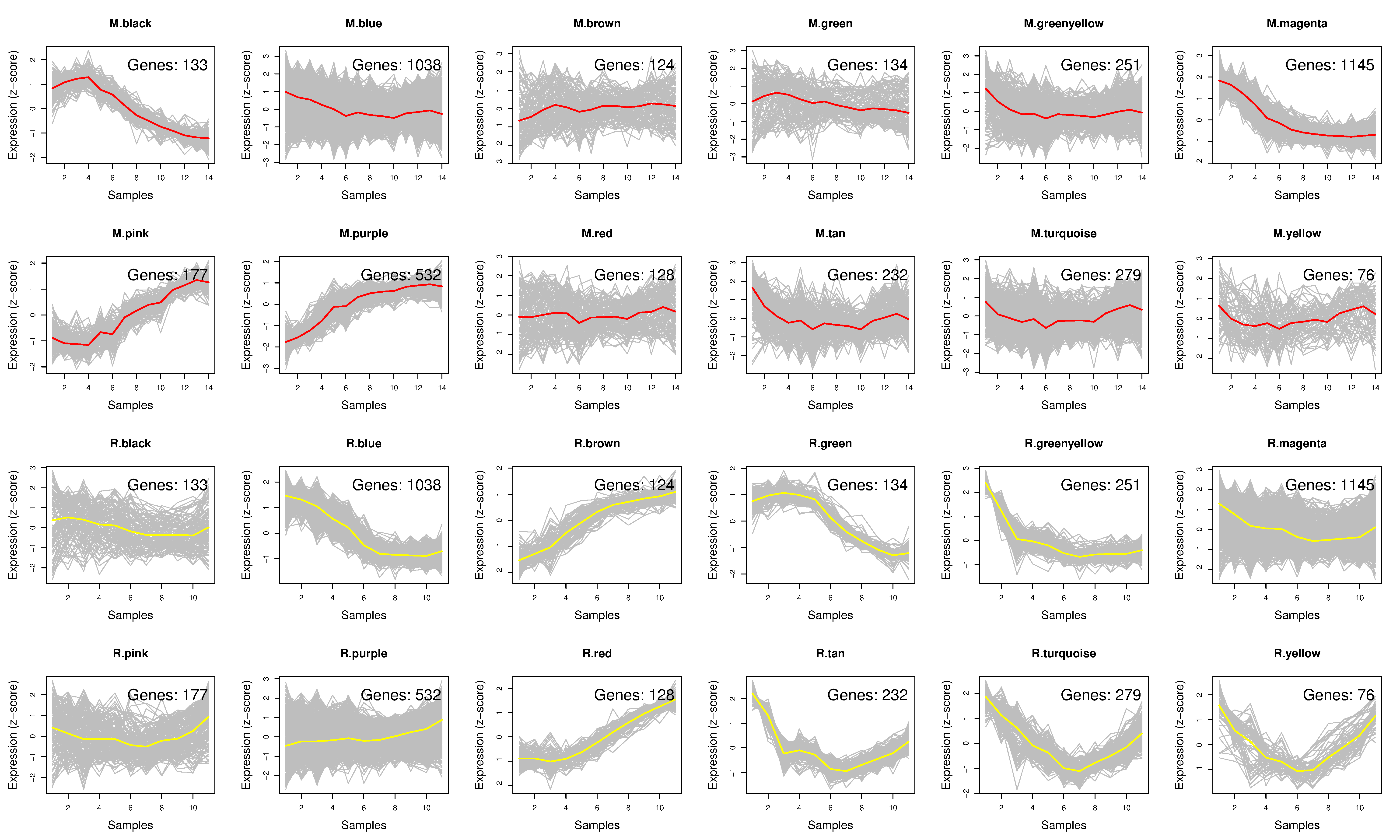

Supplement: S12 Fig — (TIF) [file pone.0140629.s012.tif]

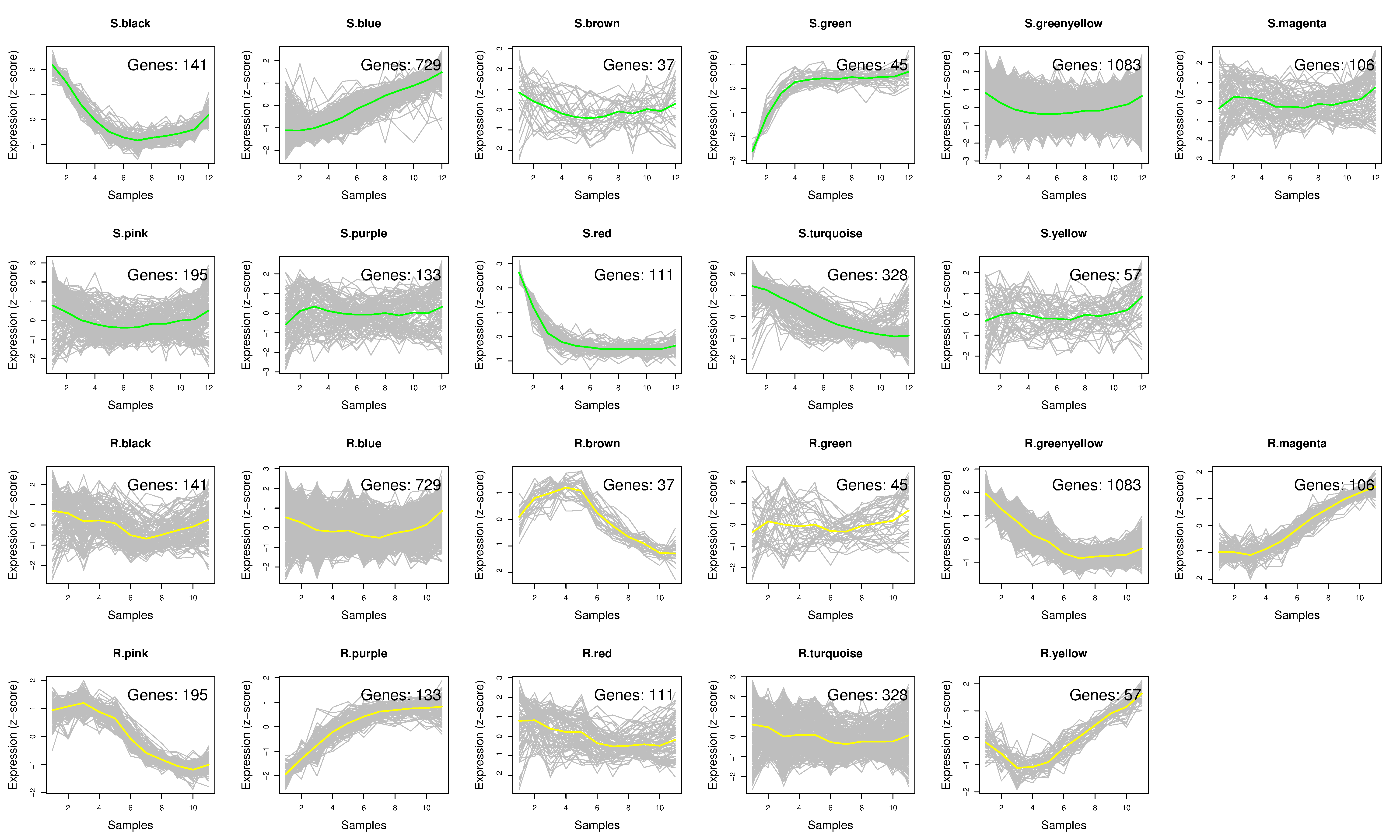

Supplement: S13 Fig — (TIF) [file pone.0140629.s013.tif]

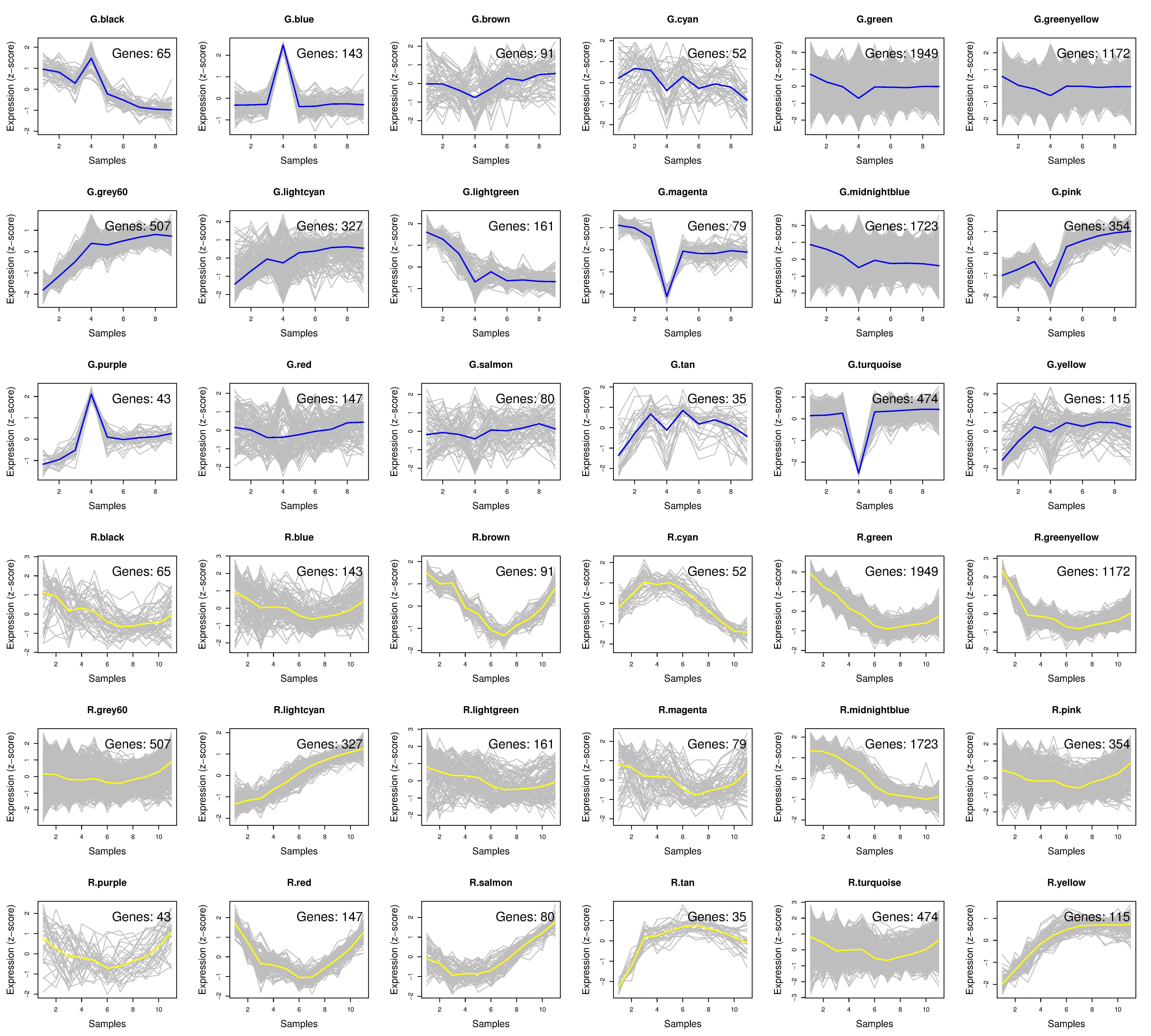

Supplement: S14 Fig — (TIF) [file pone.0140629.s014.tif]

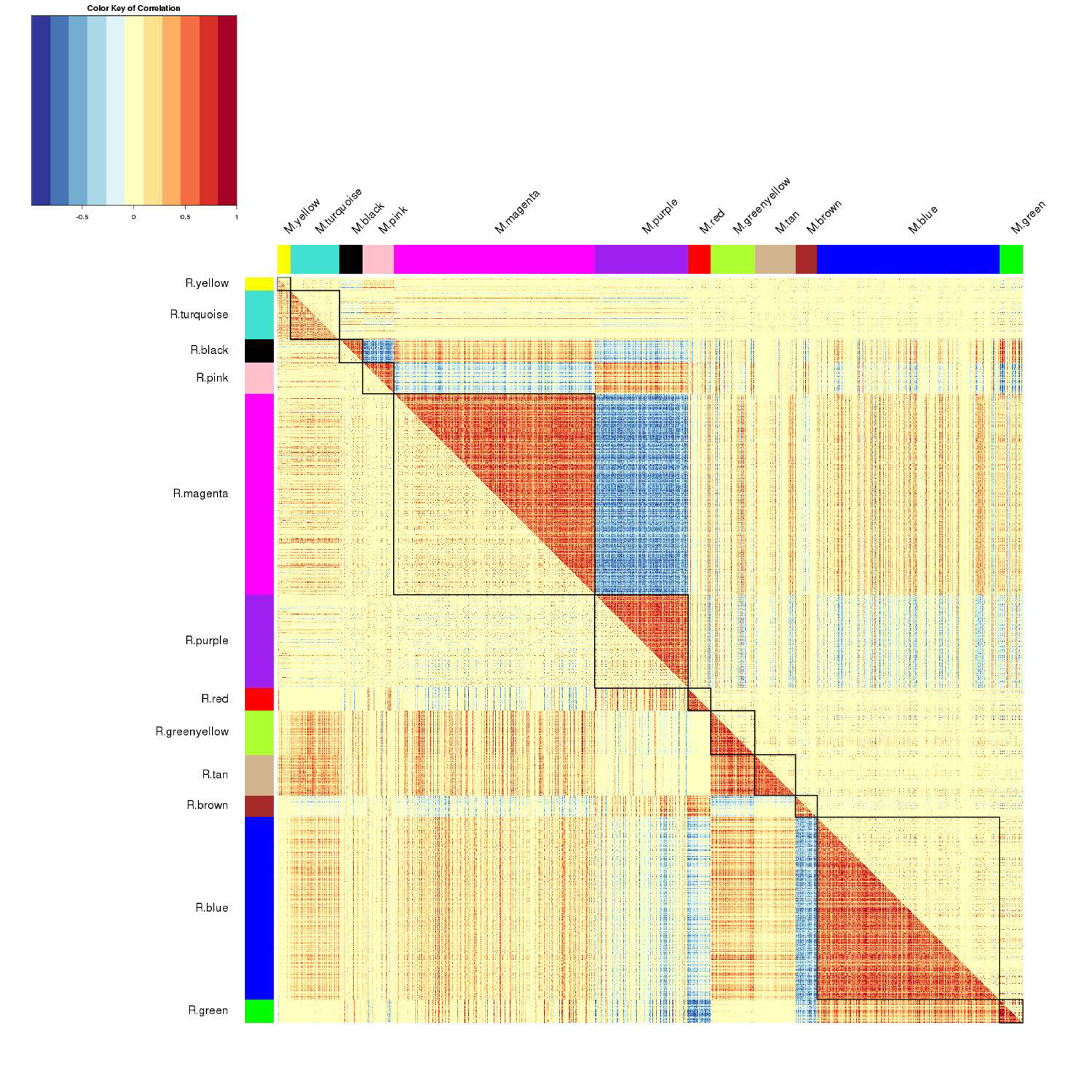

Supplement: S15 Fig — (TIF) [file pone.0140629.s015.tif]

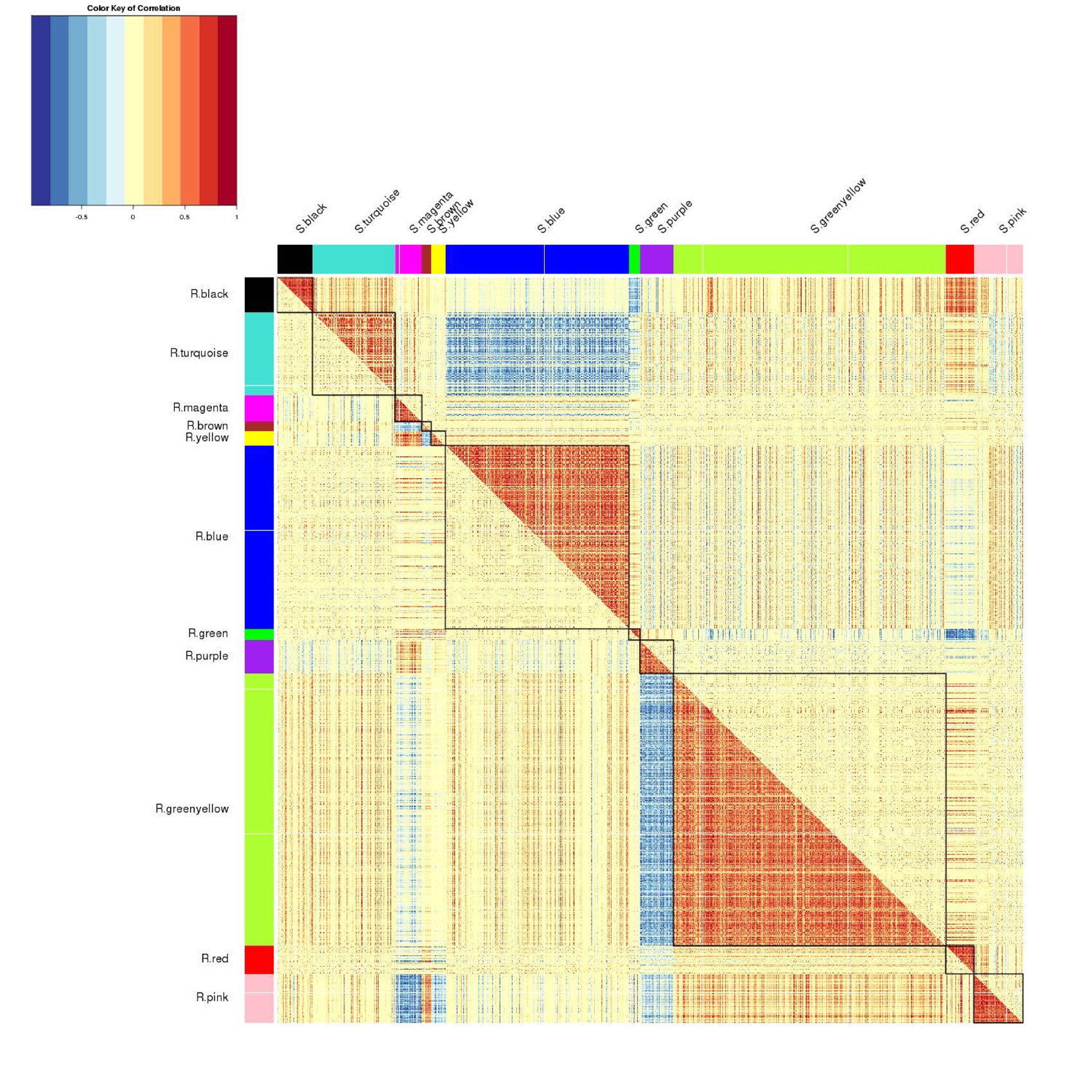

Supplement: S16 Fig — (TIF) [file pone.0140629.s016.tif]

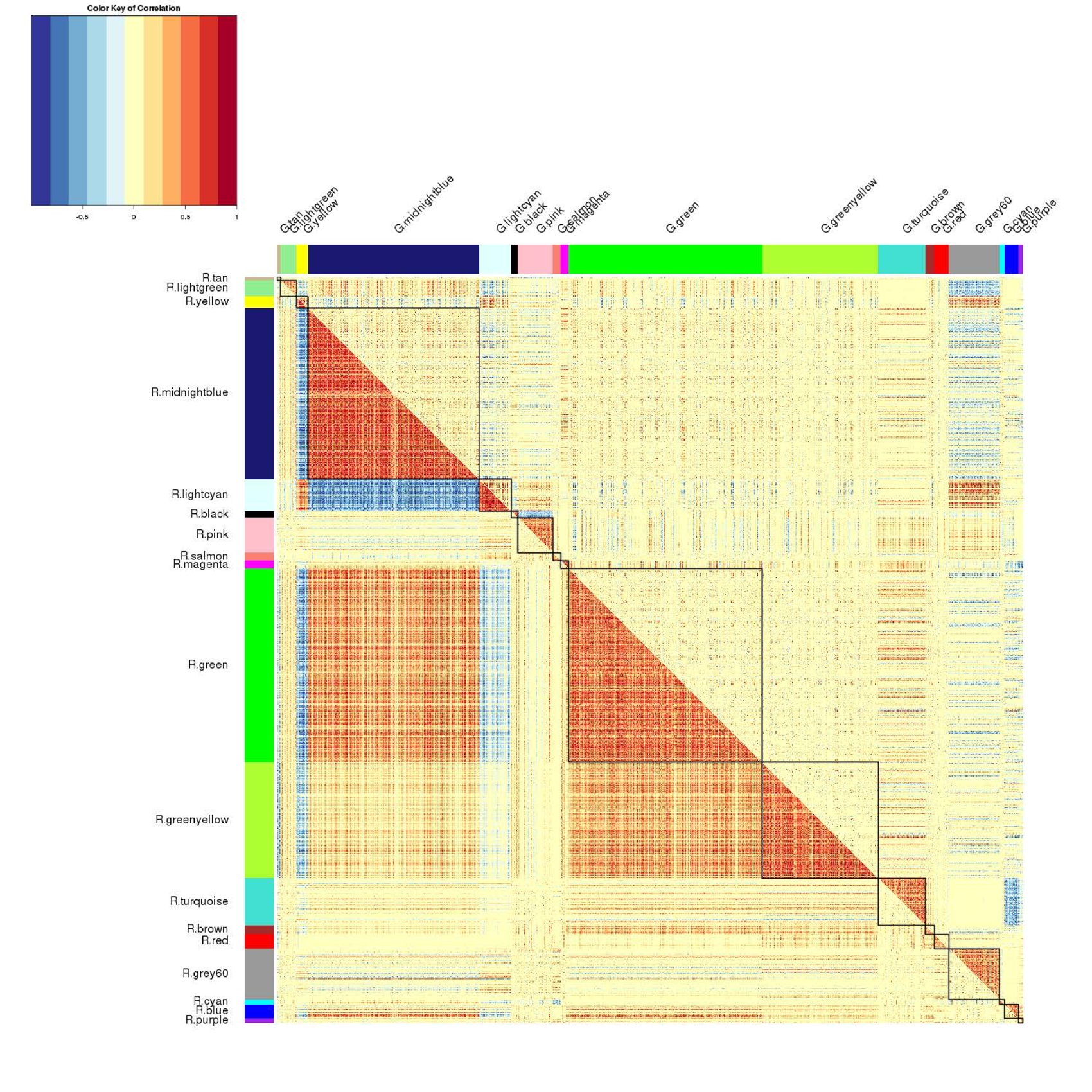

Supplement: S17 Fig — (TIF) [file pone.0140629.s017.tif]

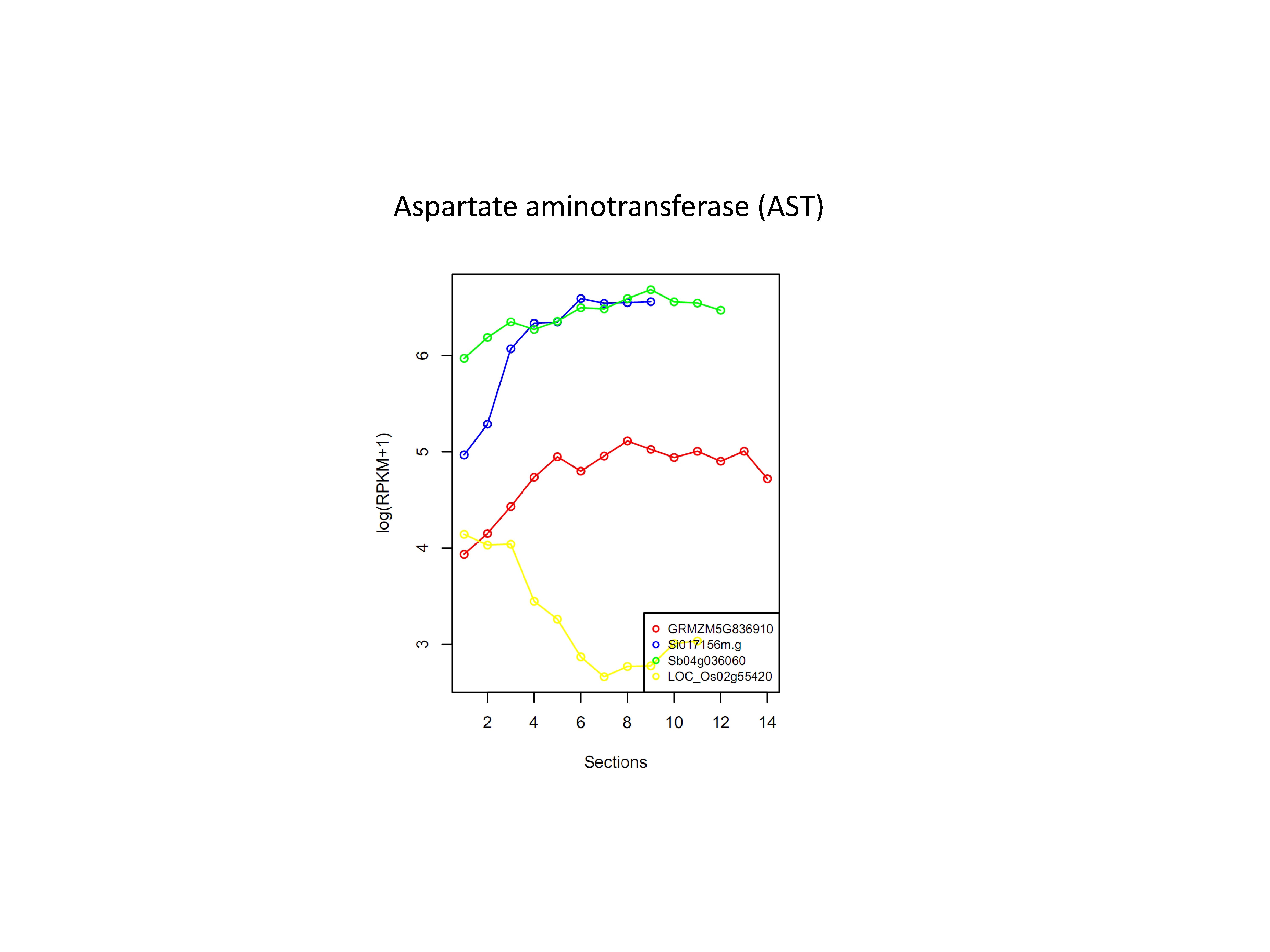

Supplement: S18 Fig — (TIF) [file pone.0140629.s018.tif]
